# Effects of nutritional counseling on dietary patterns in patients with mild cognitive impairment: insights from the BrainFit-Nutrition study

**DOI:** 10.3389/fnut.2025.1536939

**Published:** 2025-04-28

**Authors:** Etienne Hanslian, Melanie Dell’Oro, Julia K. Schiele, Farid I. Kandil, Dzenita Hasanbasic, Cirus Henn, Elmar Graessel, Julia-Sophia Scheuermann, Petra Scheerbaum, Andreas Michalsen, Michael Jeitler, Christian S. Kessler

**Affiliations:** ^1^Institute of Social Medicine, Epidemiology and Health Economics, Charité – Universitätsmedizin Berlin, Freie Universität Berlin and Humboldt-Universität zu Berlin, Berlin, Germany; ^2^Department of Internal Medicine and Nature-Based Therapies, Immanuel Krankenhaus Berlin, Berlin, Germany; ^3^Charité Competence Center for Traditional and Integrative Medicine (CCCTIM), Charité – Universitätsmedizin Berlin, Freie Universität Berlin, Humboldt-Universität zu Berlin and Berlin Institute of Health, Berlin, Germany; ^4^Brandenburg Medical School Theodor Fontane, Neuruppin, Germany; ^5^Center for Health Service Research in Medicine, Department of Psychiatry and Psychotherapy, Uniklinikum Erlangen, Friedrich-Alexander-Universität Erlangen-Nürnberg (FAU), Erlangen, Germany; ^6^Institute for General Practice and Interprofessional Care, University Hospital Tuebingen, Tuebingen, Germany; ^7^Robert Bosch Center for Integrative Medicine and Health, Bosch Health Campus, Stuttgart, Germany

**Keywords:** whole food plant-based diet, planetary health diet, vegetarian, vegan, dietary patterns, cognitive health, mild cognitive impairment, online nutritional counseling

## Abstract

**Introduction:**

This study examines the effects of a structured nutritional counseling intervention for individuals with mild cognitive impairment (MCI) via synchronized online courses conducted bi-weekly over six months.

**Methods:**

This work presents a secondary analysis of the BrainFit-Nutrition study, which explored the impacts of both 1) dietary counseling interventions (comparing a Whole Food Plant-Based (WFPB) diet with a diet based on the German Nutrition Association guidelines or Deutsche Gesellschaft für Ernährung, DGE) and 2) standardized versus individualized computer-based cognitive training, within a 2x2 factorial randomized controlled trial design for participants with MCI. While the primary outcome of the BrainFit-Nutrition study assessed the impacts of diet and cognitive training on cognitive performance, this secondary data analysis focuses on dietary habits and their changes over time. Dietary behaviors in 261 participants (52.2% female), aged between 60 and 86 years, were monitored using food frequency questionnaires at baseline (t0), post-intervention (t6), and at a 12-months follow-up (t12). Short-term (t6) and long-term (t12) dietary pattern effects were analyzed by comparing consumption frequencies across various food categories between the dietary groups, employing ANCOVAs with baseline values (t0) as covariates for exploratory analysis.

**Results:**

Throughout the intervention period, most participants in both groups maintained an omnivorous diet, with minimal shifts towards pescatarian, ovolacto-vegetarian, and vegan diets, especially in the WFPB group, which saw a minor increase in vegan and ovolacto-vegetarian participants by the end of the study. Across both dietary groups, vegetable, fruit, and whole grain consumption remained steady, with no notable intergroup differences. A decrease in meat, fish, and egg consumption was observed in both groups, with a more marked reduction in the WFPB group.

**Discussion:**

These findings suggest that while targeted dietary interventions can foster healthier dietary patterns among MCI patients, the type of dietary choices may be less impactful for individuals with MCI than participation in dietary interventions in general. Further prospective research is warranted to clarify the potential benefits of dietary adjustments on cognitive health and to refine dietary guidance tailored to this specific population.

## 1 Introduction

Growing evidence suggests an impact of dietary patterns on cognitive functions (1, 2). Established dietary regimens such as the MIND Diet (Mediterranean-DASH Intervention for Neurodegenerative Delay), MedDiet (Mediterranean Diet) and DASH Diet (Dietary Approaches to Stop Hypertension) emphasize the consumption of whole plant-based foods (2). These diets have been associated with reductions in inflammatory markers (3–6). Current findings from the German Center for Neurodegenerative Diseases (German: Deutsches Zentrum für Neurodegenerative Erkrankungen e.V., DZNE) longitudinal study suggest protective effects of the Mediterranean diet against cognitive decline and dementia (7). A recent randomized controlled pilot trial showed that comprehensive lifestyle changes, including a whole food, minimally processed plant-based diet – high in complex carbohydrates from fruits, vegetables, whole grains, legumes, seeds, and nuts, while avoiding harmful fats, refined carbohydrates, and sweeteners – may significantly improve cognitive function in individuals with mild cognitive impairment or early-stage Alzheimer’s disease (8). It is pertinent to note the potential ameliorating effects of whole food plant-based diets (WFPBD) on conditions commonly related to cognitive decline as potential risk factors, such as cardiovascular diseases, type 2 diabetes, hyperlipoproteinemia and obesity (9–13). Associations between these highly prevalent conditions and dementia have been observed (14, 15), and evidence suggests that even modest weight loss may positively influence cognitive performance (15, 16). Plant-based diets have been successfully used in intervention studies for treating type 2 diabetes (10, 11) and coronary heart disease (13, 17). Further, a randomized controlled trial (RCT) showed that a WFPBD can lead to effective and sustainable weight loss (16). Given that cardiovascular disease, type 2 diabetes mellitus, and obesity are associated with the occurrence of dementia, and a weight loss of only 2 kg can be accompanied by improved attention and memory performance (15), it seems plausible that a plant-based diet may have the potential to positively impact Mild Cognitive Impairment (MCI) symptoms.

The relevance of dietary habits for various health outcomes is well-documented. Dietary choices are associated with risks for several non-communicable chronic diseases (NCDs). Approximately 11 million premature deaths among adults could possibly be prevented by transitioning from average western dietary patterns, which often include highly processed foods rich in saturated fats, sugars, and salt, to a WFPBD (18). Data suggests that a WFPBD may be protective against a range of conditions associated with a higher risk for dementia including diabetes mellitus, arterial hypertension, other (chronic) cardiovascular disorders, and specific cancers (19–23). This protective effect is partly attributed to a reduced intake of animal proteins (24, 25). Additionally, phytochemicals, dietary fiber, and plant proteins have been investigated for their potential health benefits, including anti-inflammatory and cholesterol-lowering effects amongst others (20, 26, 27). Given that MCI may be associated with micro-inflammatory processes (28) and only plant foods and fungi can contain certain anti-inflammatory, bioactive substances such as phytochemicals and dietary fiber (26), it is worthwhile to further investigate potential neuroprotective effects of plant-based diets in well-planned clinical trials. Although fungi belong to a distinct taxonomic kingdom separate from plants, they are often included in discussions of plant-based diets due to their perceived “plant” role in society and academia in such dietary patterns. For practical purposes in this context, we will categorize fungi along with plants. There is evidence that certain plant foods such as green leafy vegetables (29), mushrooms (30), soy products (31), blueberries (32, 33), nuts (34, 35), cocoa (36) and green tea (37, 38) as well as certain herbs and spices (e.g., turmeric (39)) may exert anti-inflammatory and neuroprotective effects and may improve cognitive functions (40, 41). Increasing dietary fiber intake to approximately 34 grams per day may represent an effective strategy to counteract cognitive decline, as it has been shown to enhance specific aspects of cognitive function in older adults (42). The National Health and Nutrition Examination Survey conducted between 2011 and 2014 assessed mushroom consumption in adults aged 60 years and older. The study reported that regular mushroom intake, which provides antioxidants and vitamin D, was associated with performance on specific cognitive tests and may contribute to a reduced risk of cognitive decline (43). Furthermore, data indicated that participants consuming mushrooms performed better across multiple cognitive domains compared to non-consumers, with those consuming one or more portions per week achieving the highest scores (44). In addition, another study found that the consumption of edible mushrooms and algae was significantly associated with a lower risk of cognitive impairment in older adults (45). In a recent longitudinal study observing 92,383 adults over 28 years, daily consumption exceeding 7 grams of olive oil was linked to a 28% reduced risk of death from dementia, compared to individuals who never or rarely consumed olive oil (46).

It is important to note that adherence to a healthy plant-based diet, which includes whole grains, fruits, vegetables, nuts, legumes and tea, is associated with maintaining functional well-being in middle-aged and older adults. In contrast, diets that emphasize less healthy plant-based foods, such as fruit juices, refined grains, sugar-sweetened beverages, and sweets, have been linked to detrimental outcomes (47). Regarding the detrimental effects of unhealthy nutrition on cognitive health, there are relevant indications of associations between increased consumption of fructose, particularly through industrial sweeteners such as high fructose corn syrup, found in most processed foods, and cognitive impairment, including reduced brain volume and memory impairment, and a possible reduction in blood flow to critical brain areas such as the hippocampus and thalamus (48–50). It has been hypothesized that these regions may exhibit early metabolic decline in Alzheimer’s disease (51). On the other hand, the intake of a high quantity of vegetables and fruits seems likely to confer positive effects on cognitive function across the lifespan, with advantages particularly for the elderly (52).

While some reviews have examined potential benefits of specific micronutrients on cognitive function in individuals with MCI or dementia, the results have been inconsistent so far (53–55). This raises the question of whether a comprehensive dietary approach, rather than isolated nutrient supplementation, might be more relevant and effective for cognitive health (52, 56–60).

This secondary data analysis is part of the BrainFit-Nutrition study, a 2×2 factorial RCT involving participants with MCI (61). It utilizes longitudinal data from the nutritional intervention segment of the trial. The main objective of this analysis is to conduct a detailed examination of changes in dietary patterns at baseline (t0), six-month (t6), and twelve-month (t12) intervals measured by a food frequency questionnaire (FFQ).

## 2 Materials and methods

### 2.1 Study design

The design of the main study and its protocol have been previously published (61). In short, the main study investigated in a 2×2 factorial design whether different kinds of online nutritional group counselling and computerized cognitive training (CCT) have an impact on the cognitive abilities of patients with diagnosed MCI. In regard to the factor nutritional group counselling, the intervention comprised either a whole food plant-based diet (WFPB) or a healthy omnivorous diet based on the guidelines of the German Nutrition Society (DGE – “Deutsche Gesellschaft für Ernährung”). With respect to the computerized cognitive training (CCT), the intervention consisted of either a basic learning platform for cognitive training (basic CCT; bCCT) or an intervention focused on an adaptive self-learning platform for cognitive training (individualized CCT; iCCT).

While the CCT intervention was double-blind, the online nutritional group counselling naturally could only be single-blind, since the nutritional counselors delivering their respective intervention had to be aware of the specific guidance in order for them to provide them.

As this secondary data analysis focuses on dietary changes only, the factorial design of 2×2 groups used in the main study was collapsed into a simple 2-group design for which data were sampled over three time points (t0, t6 and t12) (61). That is, groups were distinguished hitherto only by means of the dietary intervention scheme applied in their group (WFPB vs. DGE), irrespective of the cognitive training scheme allocation.

The study procedures obtained approval from the Ethics Committee of Friedrich-Alexander-Universität Erlangen-Nürnberg’s medical faculty (Ref.: 21-318_1-B), and the study was registered at the International Standard Randomized Controlled Trial Number Registry (ISRCTN 10560738).

### 2.2 Eligibility of participants

Individuals interested in the study could schedule a screening appointment via the project homepage. During the screening, an examination of basic cognitive functions was conducted, using the Mini-Mental State Examination (MMSE) and the Montreal Cognitive Assessment (MoCA) in combination to distinguish between normal cognition, MCI, and dementia. Those who met the inclusion criteria were informed about the study and invited to participate.

Participants were eligible for inclusion in the study based on the following criteria: 1. Age 60 years or older; 2. Diagnosis of MCI, operationalized by a MoCA score of ≤24 and an MMSE score of ≥24; 3. Possession of a computer, laptop, or tablet (Windows, Linux, or MacOS) equipped with a microphone, camera, and internet access; 4. Provided informed consent.

Exclusion criteria were established as follows: 1. Complete blindness or deafness; 2. Lack of access to a personal computer, laptop, or tablet with internet access; 3. Normal cognitive function, defined as a MoCA score > 24; 4. Diagnosis of dementia, defined as an MMSE score < 24; 5. Acute depression, operationalized by a PHQ-9 score ≥ 12; 6. Other psychiatric or neurologically diagnosed diseases: psychosis, Parkinson’s disease, multiple sclerosis, strokes, substance abuse, serious brain diseases, severe vitamin B deficiency.

### 2.3 Outcomes

The primary outcome of the main study was the assessment of cognitive impairment and its progress over time as measured with the MoCA test. These results will be published elsewhere. Key additional secondary outcomes include cognitive function, evaluated by using the MMSE and a computerized cognitive test battery, measured at the t0, t6 and at a 12-months follow-up (t12).

The current exploratory study focused on the dietary behavior and its changes following the two dietary interventions between baseline, t6 and t12. Dietary behavior was assessed using the 125-item FFQ of the German Health Examination Survey for Adults (German: “Deutsches Erwachsenen Gesundheitssurvey 1,” DEGS1), modified and extended on both plant-based and animal-based foods as well as neuroprotective foods to assess the frequency at which different food groups are being consumed; for details see 2.5 (62).

### 2.4 Recruitment and randomization

The sample size was determined for the main study with the four groups of the original 2×2 design (Factor 1: WFPB vs. DGE, Factor 2: iCCT vs. bCCT) and resulted in 50 participants per group, i.e., 200 participants in total (61, 63). Since the study was conducted entirely online, participants were recruited from the general population from all over Germany. During the period between January and September 2022, 1,111 German residents aged 60 and above were screened for MCI. From the 326 positively MCI-diagnosed participants, 271 gave their written consent to participate, and were randomized into one of the four groups.

### 2.5 Survey instruments

This study used a modified version of the DEGS1 FFQ (62): of the original questionnaire, 50 items were retained, 25 were modified, and 57 items were self-designed to assess the consumption frequency of relevant plant-based and animal-based foods, as well as neuroprotective ingredients (Supplementary Table 1). While the original DEGS1 FFQ has undergone relative validation, our modified version has not yet been validated. Conducting such an FFQ validation would have required a large sample size, which was not feasible within the scope of this study. Previous research (64) highlights that validating FFQs is highly resource-intensive and demands a large number of participants, which is why, in most cases, modified FFQs are not classically validated. For this reason, we also decided against pursuing validation to maintain the feasibility and focus of this investigation. The original DEGS1 FFQ was tested among participants of the DEGS1 study, demonstrating reasonable validity in assessing the dietary patterns of German adults (64).

Data presented in this work focus on food group recommendations from official German dietary guidelines (e.g., vegetables, fruits, legumes and whole grains; animal products such as milk, certain dairy products, meat, fish, and eggs), plant-based alternatives (including plant-based milk and plant-based spreads), as well as the consumption frequency of specific food items regarded as protective of patients’ brain health (e.g., berries, leafy vegetables, broccoli, mushrooms, seeds, unsalted nuts and walnuts, herbs, and green tea). The analysis also highlights items potentially harmful to mental health (e.g., fast food, snacks, and sweets such as fried sausages, savory snacks and chips, pastries, sugary spreads, chocolate and chocolate bars, sweets, and sugared and light beverages, and alcoholic drinks). An additional question assessed the frequency of cooking, asking participants how often per week they prepare a hot meal (lunch or dinner) for themselves from basic ingredients/fresh food, with response options ranging from ‘daily’ to ‘never’.

As the DEGS1 FFQ collects data (for most items) about both the consumption frequency per month/week/day as well as the amount per meal (in pre-defined servings sizes), data was collapsed into servings per month/week/day to allow for easy comparison.

Classification of participants into omnivore, pescatarian, ovo-lacto vegetarian and vegan followed participants’ statements in the DEGS1 survey. Participants stating consumption of red meat, poultry, cold cuts or meat-based fast food at all were classified as “omnivores,” independent of the amount or consumption frequency of these food items. Participants who were not omnivores, but consumed fish were labeled as” pescetarians.” Participants who were consuming neither meat-based nor fish-based dishes were labeled as “ovo-lacto vegetarians” if they consumed eggs, animal milk, or produces derived from animal milk. If they abstained from consuming any meat, fish or food produced by animals, they were finally classified as “vegans.”

### 2.6 Data collection

FFQ data were collected online at baseline, t6 and t12. Data were managed using REDCap, a web-based platform hosted at Charité Berlin. This system was used to provide the FFQ, which participants accessed through a personalized link provided via email.

Adherence to the online nutritional group counselling was tracked in a table using pseudonyms, with participants encouraged to communicate cancelations in advance.

### 2.7 Statistical analysis

As mentioned above, the current study focuses on dietary changes only, the factorial design of 2×2 groups used in the main study was collapsed into a simple 2-group (WFPB vs. DGE) design with three time points (t0, t6 and t12) (61). Both immediate and long-term effects were analyzed between groups using separate analyses of covariance (ANCOVAs). To estimate immediate effects, for each food category differences between the groups at t6 were compared using an ANCOVA, with baseline (t0) values for that food category as a covariate. To estimate corresponding long-term effects, this procedure was then repeated between t12-values for the two groups and baseline values as covariates (61). However, to estimate immediate (t0 to t6) and long-term changes (t0 to t12) in the diet type, relative frequencies were compared between groups by means of a logistic regression for t6 values or t12 values, respectively, with group and t0 values as covariates.

Next, to these between-group comparisons, we also analyzed intragroup changes between t0 and t6 (immediate effects) as well as between t0 and t12 (long-term effects) by means of missing values, which were imputed using the MICE algorithm for multiple imputations for the secondary analyses presented in this study. Imputation and all statistical analyses were performed using custom written scripts in Python (version 3.12, along with the tools statsmodels, scipy and sklearn).

All analyses were conducted on an exploratory level only. Thus, differences for which *p* < 0.05 were obtained are regarded as interesting as possible outcomes for upcoming studies, but the resultant differences are not regarded as significant in the sense of confirmatory evidence. Consequently, all *p* < 0.05 are interpreted only as so called “exploratively” and thus labeled as interesting for a future study, but not as significant.

### 2.8 Nutritional concepts and interventions

#### 2.8.1 Online nutritional group counselling

Detailed information on the design of the online nutritional group counselling has been published previously (61). Nutritional courses were conducted online via Microsoft Teams, an online meeting software. Access was facilitated through webMODYS, a participant management system hosted on a secure server, which emailed participants a direct access link for pseudonymized entry, requiring no additional registration or software installation. Participants were required to have a stable internet connection, as well as a microphone and video camera for participation.

The online nutritional group counselling sessions were based on either a WFPB diet or a DGE diet, spanning 12 sessions over six months (each lasting 90 min and held biweekly). Each session was attended by 10–25 participants. The sessions were conducted on the same evening, consecutively, and were led by two distinct course instructors professionally trained in nutrition. Table 1 provides a general overview of the 12 sessions for both intervention groups, which were standardized in their structure. After 6 months participants were free to keep the new nutritional instructions or go back to their normal diet in an open phase until t12.

**Table 1:** Standardized curriculum for both nutritional intervention groups.

Time	Session 1	Session 2	Session 3	Session 4	Session 5	Session 6	Session 7	Session 8	Session 9	Session 10	Session 11	Session 12
1-5 min	Mindful minute – Joining - “Check in”											
20 min	Welcome	Reflection – Pending questions – Discussion on the process of behavioral changes										
20 min	Nutrition Basics 1	Nutrition Basics 2	Practical applications in everyday life	Kitchen practice in theory; Healthy cooking and baking	Kitchen practice“virtual” buffet - Live cooking	Influence of nutrition on health; Macronutrient protein	Macronutrient carbohydrates: Dietary fibers; Microbiome	Fats, nuts, seeds; Drinks (alcohol, tea, coffee)	Special nutrients: Phytochemicals, spices, herbs and age-specific nutrition	Circadian factors of nutrition; Periodic fasting	Mindfulness:Conscious eating and sensory exercise; Emotional eating	Conclusion; Appreciation; Repetition; Evaluation
5 min	Short break											
30 min	Practical application - Practical transfer											
	Tips for grocery shopping and recipes	Goal setting and behavior change	Day and meal planning	Own recipe creation for kitchen practice	Food studies; Exchange of optimal food items	Combining proteins; Reflection on goals	Whole grains; Cereals; Gluten free	Healthier recipes for fast food	Superfoods	Planning your own day	Questionnaire: Mindful eating	New goal setting for “free phase”
10 min	Next steps - “homework” - conclusion											

The sessions for both groups were standardized in their structure with the initial two sessions focusing on introducing the fundamentals of recommendations. These sessions also served as an opportunity for participants to acquaint themselves with technical aspects like group rooms, microphone usage, and video settings. Sessions 3–5 shifted toward practical application, where participants learned about new food items, their preparation, and proper storage. The sixth session delved into the rationale and scientific foundation behind the provided recommendations. From sessions 7–9, the course focused on different nutrients and recommended foods within specific groups, deepening participants’ nutritional knowledge, including micronutrients and age-specific guidelines. Addressing the “how and when” of eating, two additional sessions were dedicated to mindful eating and circadian factors.

#### 2.8.2 Counselling focusing on a WFPB diet

The WFPB diet concept, systematically taught to the participants randomized into this group, primarily consisted of vegetables, whole grains, legumes, fruits, nuts, and seeds, without restricting energy intake (see Table 2). This diet was recommended for regular consumption. Furthermore, participants were encouraged to regularly consume specific foods and nutrients believed to have a beneficial impact on cognitive functions based on current scientific evidence, such as green leafy vegetables (29), mushrooms (30), citrus fruits (65), soy products (31), blueberries (32), nuts (34), turmeric (39), green tea (37) and omega-3 fatty acids (57).

**Table 2:** Recommended foods with potential beneficial impact on cognitive functions.

Recommended daily	To be avoided
Whole grains (e.g., rice, pasta, millet, quinoa)	Highly processed foods
Blueberries and other berries	Sugary drinks, white sugar, and refined grains
Broccoli (raw, blanched, steamed)	Alcohol consumption
Green leafy vegetables (e.g., spinach, chard, bok choy)	Food high in saturated fats (e.g., butter, cheese, cream, and meat)
Spices and herbs (e.g., turmeric, coriander, saffron)	Trans-fats from deep-fried food (e.g., chips, fries, bakery items, fast food)
Mushrooms	Processed meats like sausage
Green tea, dark chocolate (80% cocoa)	
Omega-3 rich oils such as flaxseed oil	

Concurrently, participants were advised to abstain from animal products in their diets during the intervention period due to their proinflammatory potential and to avoid consuming highly processed foods (66) (Table 2).

#### 2.8.3 Counselling focusing on a DGE diet

Participants randomized to this group received recommendations according to the official guidelines of the DGE diet for healthy eating (67). They were systematically encouraged to establish an omnivorous diet rich in vegetables, fruits, and whole grains, including moderate intake of animal products, such as fish, poultry, red meat, eggs and dairy products (Table 3). The DGE group was also advised to prioritize fresh, unprocessed foods and to minimize their consumption of saturated fatty acids, sweetened drinks, and highly processed foods.

**Table 3:** Daily nutritional recommendations for WFPB and DGE interventions.

Category	WFPB recommendations	DGE recommendations
Vegetables	At least 3 portions (plus one tbsp. of sea vegetables/algae)	3 portions
Fruits	2 portions	2 portions
Cereals	3 to 4 portions, whole grains	4 portions, whole grains including potatoes
Nuts and seeds	1 to 2 portions	Mentioned as an alternative to one fruit portion
Legumes	1 portion	Not specified
Plant oil	2 to 3 tbsp (especially flax seed oil)	1.5 to 2 tbsp (1 tbsp plant oil plus 15–30 g butter or margarine)
Animal fats	Not recommended	1.5 to 2 tbsp
Milk products	Not recommended	3 portions
Meat, poultry, fish, eggs	Not recommended	300–600 g meat as well as 1–2 servings of fish (fatty, low-fat), 3 eggs per week, and 3 servings of sausage (30 g each)
Milk alternatives	1 to 3 portions	Not specified
Sweets, fried foods, fast foods	Not recommended	Maximum 1 portion
Neuroprotective foods	Daily (e.g., walnuts, flaxseed oil, berries, green leafy vegetables, herbs)	Not specified

DGE, German Nutrition Association (German: Deutsche Gesellschaft für Ernährung); WFPB, Wohle-Food Plant-Based.

#### 2.8.4 Food boxes

Every participant received six different monthly food box deliveries by regular post during the period of the study interventions. The boxes for the WFPB group contained selected wholesome, neuroprotective and durable food items like wholegrain pasta, green tea, nuts, grains, plant oils and healthy snacks like sugar-free chocolate. The delivery boxes for the DGE group contained a selection of staple foods likely to exert generally beneficial health effects (e.g., whole grain, plant-based oils or nuts/seeds, sugar alternatives, other minimally processed foods and vegetarian alternatives). These food boxes, serving as sample packages, were intended to vividly introduce the food items discussed in each session. They were meant to facilitate a transfer from theoretical knowledge to practical application, thereby supporting motivational behavior change and encouraging participants to explore new and beneficial food items. Neutral packaging was chosen to eliminate bias and ensure that participants’ perceptions and behaviors were not influenced by advertising. This approach allowed for a more accurate assessment of the food items based purely on their quality and appeal, not their market identity. The detailed food items of the six food boxes can be found in Supplementary Table 2.

#### 2.8.5 Adherence to dietary recommendations

The counselling sessions incorporated interactive methods and diverse learning materials, including structured handouts, specific recipes, and reflections on participants’ nutrition experiences for exchange rounds. The nutritional content was introduced gradually, with small steps to encourage personal involvement and to enhance participant attention, motivation, and commitment over the 6-months course. The participants received recipes and recommendations to incorporate these foods into daily life.

To ensure the regular integration of the recommended food groups, after six sessions, participants assessed their integration through a reflection quiz in which they rated their adherence to dietary recommendations and identified areas for improvement in their eating habits. Participants rated their adherence to recommended intakes on a scale from “do not implement” to “fully implement,” indicating how successfully they had incorporated these recommendations into their lifestyle. The reflection tool also prompted participants to identify successes, challenges, and areas for growth concerning their dietary habits. Further, it asked them to set future goals and define the steps needed to achieve them, thereby supporting continuous improvement and personal development in dietary practices. This structured reflection process aimed at enhancing the effectiveness of the dietary interventions by fostering greater self-awareness and accountability.

#### 2.8.6 Didactical and methodical concept

As outlined in Table 4, the course adopted the Health Approach Process Assessment (HAPA) model by Schwarzer, focusing on motivation and volition phases with self-efficacy as the key component (68). Risk awareness was increased by linking dietary factors with adverse health outcomes such as dementia. Outcome expectancy was enhanced through the demonstration of neuroprotective food benefits. The instructional approach integrated resource-oriented communication and motivational interviewing, to boost engagement without pressure. Reflective practices and goal setting, as part of the ongoing behavior change process, were employed to promote sustainable dietary changes (69, 70). Participants were guided to set and review their nutritional goals every six sessions, encouraging long-term health improvements.

**Table 4:** Overview of the course methodology.

Component	Description
Model	Health Approach Process Assessment (HAPA)
Phases	Motivation and volition
Key feature	Self-efficacy expectation
Behavioral determinants	Risk awareness and outcome expectancy
Trigger for risk awareness	Influence of food factors on dementia, MCI, and cardiovascular diseases
Positive outcome expectancy	Demonstrated by neuroprotective food items and effective nutritional changes
Supportive techniques	Sharing successful experiences, focusing on small-step successes, addressing individual obstacles, providing practical information
Communication style	Resource-oriented and positive
Motivational strategy	Motivational interviewing
Behavior change process	Initiated in course 2, involving reflection rounds and scaling questions
Goal setting	Participants set and evaluate realistic nutritional goals over 6 sessions

## 3 Results

A total of 1,111 patients were screened for the main study of which 326 were found eligible and 271 gave their written informed consent and were enrolled in the study and randomized in the four groups. However, only 270 participants completed the baseline visit, leading to their inclusion in the analyses [WFPB + iCCT (*n* = 65), WFPB + bCCT (*n* = 71), DGE + iCCT (*n* = 64), and DGE + bCCT (*n* = 70)]. For the data analysis presented here, patients were pooled into a WFPB group (*n* = 136) and a DGE group (*n* = 134). Of these, *n* = 132 of the WFPB and 129 of the DGE group filled out the FFQ at baseline. The numbers dropped to 101 and 85 for WFPB and 104 and 97 for DGE for the t6 and t12 visit, respectively. The mean age of the participants was 70.8 ± 6.9 years, with a range from 60 to 86 years. Slightly more than half of the 270 participants in the study were women, accounting for 52.2% of the sample. Nearly a third of the participants (29.6%) resided by themselves. More than half (58.9%) possessed a high educational background, and most of the participants (71.9%) were retired or not currently employed. The mean MoCA score was 22.4 ± 1.6, and the mean MMSE score was 27.5 ± 1.6, indicating a reduced cognitive function consistent with MCI.

The distributions between the two intervention groups—WFPB and DGE—showed no relevant differences in age, gender, living situation, education level, employment status, or cognitive skills. The characteristics of individuals with MCI in the overall sample and intervention groups can be found in Table 5.

**Table 5:** Characteristics of participants with MCI in the overall sample and intervention groups.

**Variable**		**Overall Sample (*N* = 270)** ***M* (*SD*) / *n* (%)**	**WFPB (*N* = 136)** ***M* (*SD*) / *n* (%)**	**DGE (*N* = 134)** ***M* (*SD*) / *n* (%)**
Age	Years	70.8 (6.9)	70.3 (6.4)	70.2 (7.3)
Sex	Female	141 (52.2)	72 (52.9)	69 (51.5)
	Male	129 (47.8)	64 (47.1)	65 (48.5)
Household type	Single household	80 (29.6)	35 (25.7)	45 (33.6)
Education (ISCED)	High	159 (58.9)	75 (55.1)	84 (62.7)
	Medium	104 (38.5)	58 (42.6)	46 (34.3)
	Low	7 (2.6)	3 (2.2)	4 (3.0)
Employment	Employed	76 (28.1)	35 (5.7)	41 (30.6)
Cognitive skills	MoCA	22.4 (1.6)	22.3 (1.7)	22.4 (1.5)
	MMSE	27.5 (1.6)	27.3 (1.7)	27.6 (1.6)

Age and cognitive skills are described as mean (M) and standard deviation (SD). For all other variables, absolute and relative frequencies are shown. (ISCED, International Standard Classification of Education, *n* = Count).

Of the 270 patients initially enrolled in the study 266 started the online nutritional courses (131 in the WFPB group and 135 in the DGE group), 42 dropped out of the courses very early on, leaving 224 patients participating in the courses, 108 in the WFPB and 116 in the DGE group. Of the 108 patients in the WFPB group, 78 (72.2%) took part in at least half (6 of 12) course units and are therefore considered adherent. 56 participants (51.9%) even took part in at least three quarters of the course units (9 of 12). There was a comparable frequency in the DGE group, where 88 of the 116 (75.9%) patients participated in at least half and 65 (56.0%) took part in at least three quarters of the course units.

### 3.1 FFQ longitudinal data

The study analyzed behavioral changes in participants’ diets by comparing dietary changes within and between the two groups (WFPB and DGE) over a 12-month period. Detailed descriptive statistics (group means and standard deviations or absolute and relative frequencies) for all 132 FFQ items at all three visits, along with the results of the statistical evaluation (ANCOVA) for changes between t0 and t6 and t0 and t12, respectively, can be found in Supplementary Table 3. Dietary changes within the groups are listed in Supplementary Table 4.

### 3.2 Nutritional changes

The distribution of diet types at various time points is shown in Table 6. At t0, the majority of participants in both groups followed an omnivorous diet. Specifically, 95.5% (*n* = 126) of participants in the WFPB group and 94.6% (*n* = 122) in the DGE group reported being omnivores. Smaller percentages reported following pescatarian (WFPB: 2%, *n* = 2; DGE: 3.9%, *n* = 5), ovolacto (WFPB: 2,3%, *n* = 3; DGE: 0.8%, *n* = 1), or vegan diets (WFPB: 0.8%, *n* = 1; DGE: 0.8%, *n* = 1). At t6, the proportions of diet types remained largely consistent between the groups. The WFPB group had 93.9% (*n* = 124) omnivores, 1.5% (*n* = 2) pescatarians, 3% (*n* = 4) ovolacto vegetarians, and 1.5% (*n* = 2) vegans, whereas the DGE group had 95.3% (*n* = 123) omnivores, 3.1% (*n* = 4) pescatarians, 1.6% (*n* = 2) ovolacto-vegetarians, and no vegans. At t12, the trend remained quite stable with 92.4% (*n* = 122) omnivores in the WFPB group and 95.3% (*n* = 123) omnivores in the DGE group. The percentage of participants adhering to a vegan diet increased to 2.3% (*n* = 3) in the WFPB group, while the DGE group still had 0.8% (*n* = 1) vegans. The numbers of the pescatarians were the same as t6. The percentage of participants adhering to a ovolacto diet increased to 3.8% (*n* = 5) in the WFPB group, while the DGE group only had 0.8% (*n* = 1).

**Table 6:** Changes in diet type.

Diet type	**At baseline (t0)**		**After 6 months (t6)**		**After 12 months (t12)**	
	**WFPB group**	**DGE group**	**WFPB group**	**DGE group**	**WFPB group**	**DGE group**
Omnivore	126 (95.5%)	122 (94.6%)	124 (93.9%)	123 (95.3%)	122 (92.4%)	123 (95.3%)
Pescatarian	2 (1.5%)	5 (3.9%)	2 (1.5%)	4 (3.1%)	2 (1.5%)	4 (3.1%)
Ovolacto	3 (2.3%)	1 (0.8%)	4 (3.0%)	2 (1.6%)	5 (3.8%)	1 (0.8%)
Vegan	1 (0.8%)	1 (0.8%)	2 (1.5%)	0 (0.0%)	3 (2.3%)	1 (0.8%)

Distribution in *n* (%) for the four different diet types of omnivore, pescatarian, ovolacto-vegetarian and vegan diet at the visits t0 (baseline), t6 (after 6 months) and t12 (after twelve months).

### 3.3 Vegetables, fruits, legumes and whole grains

**Figure 1:**
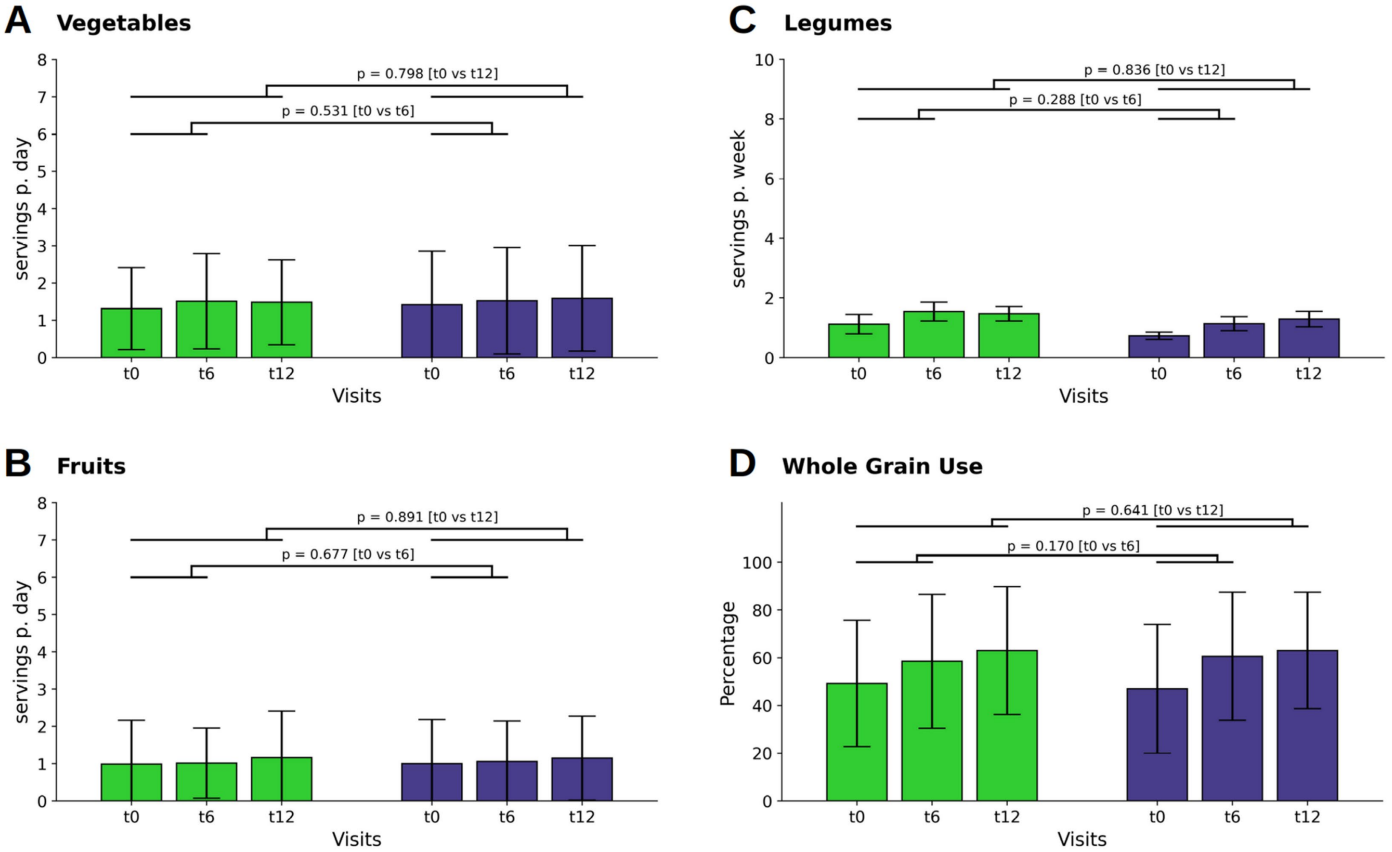
Changes in consumption of vegetables **(A)**, fruits **(B)**, legumes **(C)** and the percentage of whole grain usage in respect of the individual total grain consumption **(D)**. Bars and whiskers indicate mean and standard deviation, respectively, for the visits t0 (baseline), t6 (after 6 months) and t12 (after twelve months). Green and blue bars depict frequencies in the WFPB and the DGE group, respectively. Horizontal bars indicate results (*p* = *p*-values) of the ANCOVAs calculated for t6 and t12 values, respectively, with t0 values serving as covariates in both comparisons.

Analyses within each group showed that both WFPB and DGE participants maintained consistent intake levels of vegetables and fruits throughout the whole study period, with no notable changes observed across all time points (Figures 1A,B).

Legume intake increased slightly at t6, following the active dietary intervention, in both the WFPB group (M ± SD: 0.06 ± 0.35; *p* = 0.049) and DGE group (0.06 ± 0.21; *p* = 0.002) without relevant difference between the groups at t12 (Figure 1C).

Whole grain consumption was balanced between the groups at baseline. At t6 there was an increase in whole grain intake in both groups (WFPB: M ± SD: 9.28 ± 24.02; *p* < 0.001; DGE M ± SD: 13.57 ± 21.12; *p* < 0.001), with a further and similar increase in both groups by t12 (WFPB: M ± SD: 13.79 ± 25.50; DGE: M ± SD: 16.01 ± 23.15; *p* < 0.001) However, the between-group differences of all above mentioned parameters were not relevant (Figure 1D).

### 3.4 Animal products

**Figure 2:**
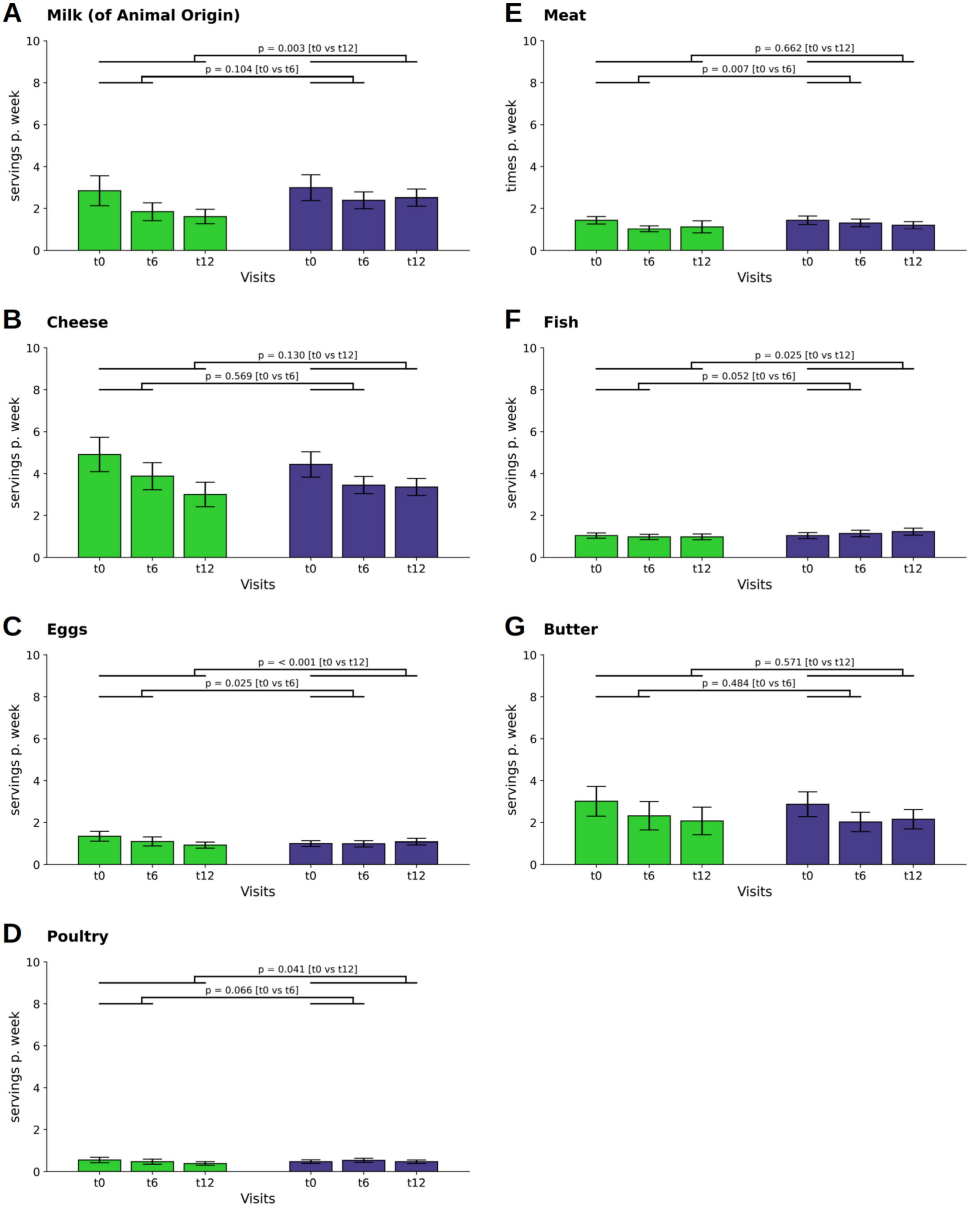
Changes in consumption of food from animal sources. Consumption of milk **(A)** and cheese **(B)**, eggs **(C)**, poultry **(D)**, fish **(F)** and butter **(G)** are depicted as servings per day or week, that is the combination of individual frequencies with individual serving sizes. In contrast, the consumption of meat **(E)** is given as times per week. Bars and whiskers indicate mean and standard deviation, respectively, for the visits t0 (baseline), t6 (after 6 months) and t12 (after twelve months). Green and blue bars depict frequencies in the WFPB and the DGE group, respectively. Horizontal bars indicate results (*p* = *p*-values) of the ANCOVAs calculated for t6 and t12 values, respectively, with t0 values serving as covariates in both comparisons.

**Figure 3:**
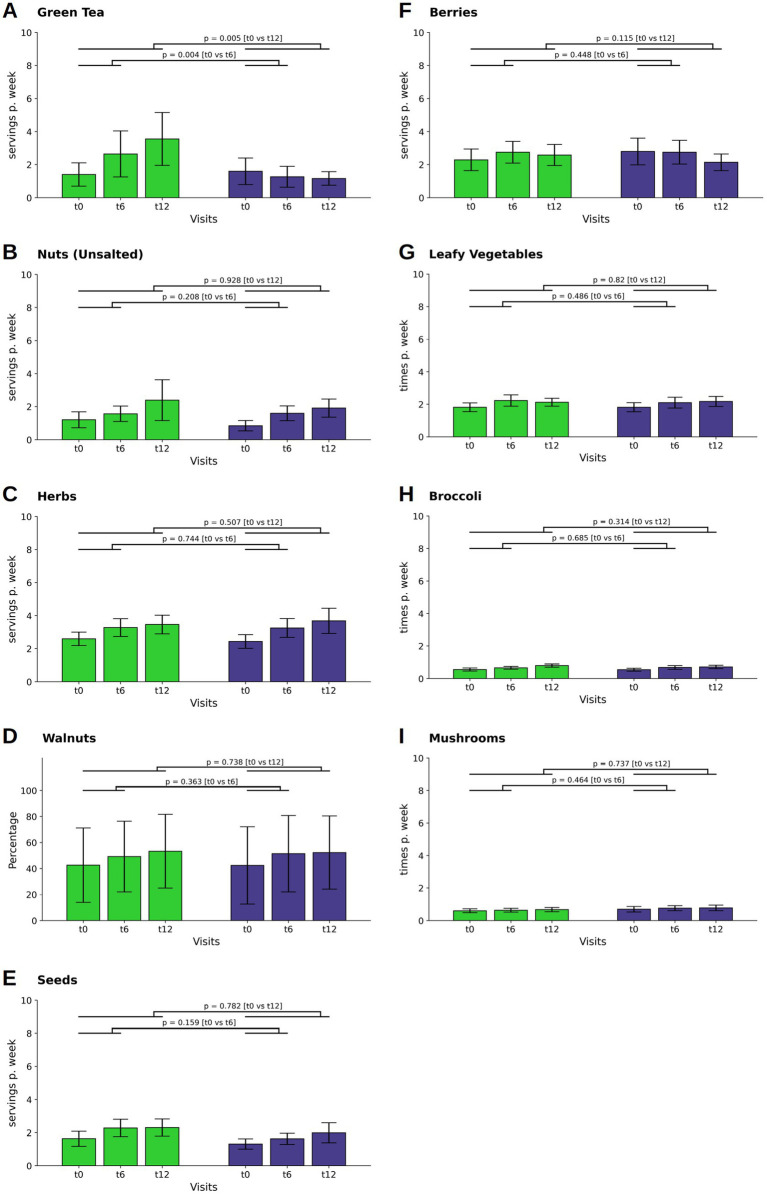
Changes in the consumption of neuroprotective food items. The consumption of green tea **(A)**, nuts **(B)**, herbs **(C)**, seeds **(E)**, and berries **(F)** and are depicted as servings per day or week, that is the combination of individual frequencies with individual serving sizes, whereas the consumption of leafy vegetables **(G)**, broccoli **(H)** and mushrooms **(I)** are given as times per day or week, In contrast, walnut consumption **(D)** is presented as the percentage of total nut consumption. Bars and whiskers indicate mean and standard deviation, respectively, for the visits t0 (baseline), t6 (after 6 months) and t12 (after twelve months). Green and blue bars depict frequencies in the WFPB and the DGE group, respectively. Horizontal bars indicate results (*p* = *p*-values) of the ANCOVAs calculated for t6 and t12 values, respectively, with t0 values serving as covariates in both comparisons.

There was a difference in the decline of milk consumption between the two groups at t12 (*p* = 0.003; WFPB: M ± SD: 0.23 ± 0.35; DGE: M ± SD: 0.36 ± 0.4 Figure 2A). Cheese consumption decreased in both groups during the first six months of the dietary intervention and decreased even further in the WFPB group by t12 (WFPB: M ± SD: −0.15 ± 0.70; *p* = 0.017 at t6 and M ± SD: −0.27 ± 0.68; *p* < 0.001 at t12; DGE: M ± SD: −0.14 ± 0.49; *p* = 0.001 at t6 and M ± SD: −0.15 ± 0.50; *p* = 0.001 at t12; Figure 3B). However, the differences between the groups were not statistically significant.

Egg consumption decreased in the WFPB group (M ± SD: −0.06 ± 0.15; *p* < 0.001 at t12), leading to a difference between the groups (*p* < 0.001; WFPB: M ± SD: 0.13 ± 0.15; DGE: M ± SD: 0.16 ± 0.16 at t12, Figure 2C). There was also a reduction in the consumption of poultry in the WFPB group at t12 (M ± SD: −0.02 ± 0.10; *p* = 0.008) leading to a difference between the two groups (*p* = 0.041; WFPB: M ± SD: 0.05 ± 0.09; DGE: M ± SD: 0.07 ± 0.08 at t12, Figure 2D).

A difference in the reduction of meat consumption was observed at t6 (*p* = 0.007; WFPB: M ± SD: 0.15 ± 0.14; M ± SD: DGE: 0.19 ± 0.19) but was not maintained throughout the observational phase (*p* = 0.662; WFPB: M ± SD: 0.16 ± 0.29; M ± SD: DGE: 0.17 ± 0.17 at t12, Figure 2E). Fish consumption showed an increase at t12 in the DGE group (M ± SD: 0.03 ± 0.14; *p* = 0.031), leading to a difference between the groups (*p* = 0.025; WFPB: M ± SD: 0.14 ± 0.14; DGE: M ± SD: 0.18 ± 0.17 at t12, Figure 2E).

Butter showed a decline in both groups (WFPB: M ± SD: −0.10 ± 0.35; *p* = 0.001 at t6 and M ± SD: −0.13 ± 0.39; *p* < 0.001 at t12; DGE: M ± SD: −0.12 ± 0.36; *p* < 0.001 at t6 and M ± SD: −0.10 ± 0.46; *p* = 0.013 at t12) without a significant intergroup difference (Figure 2G).

### 3.5 Plant-based alternatives

**Figure 4:**
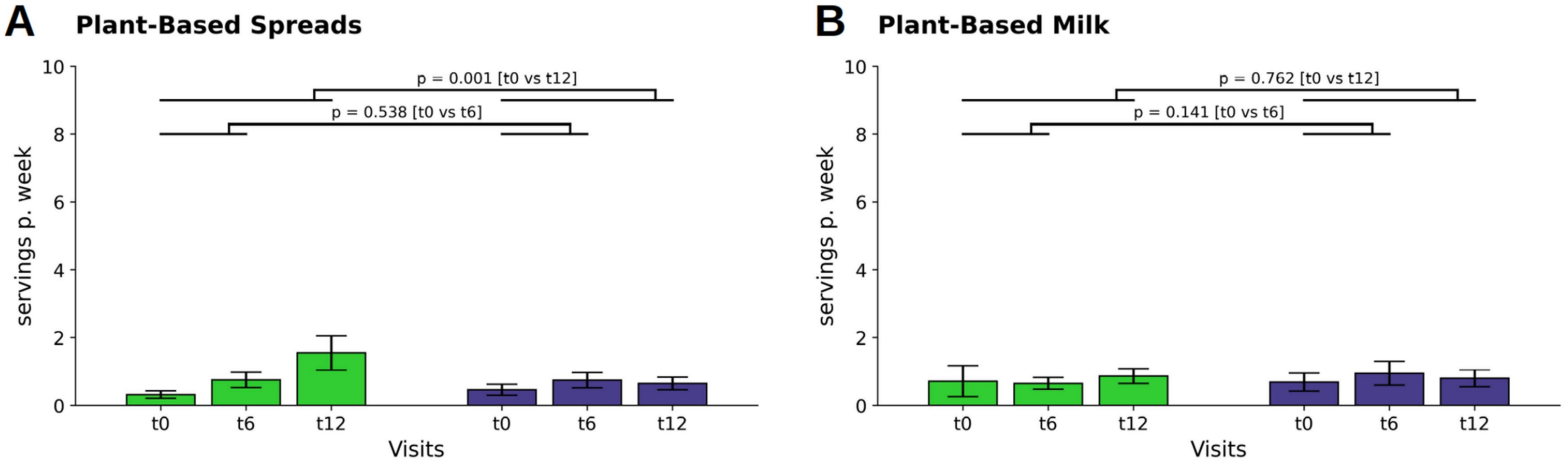
Changes in consumption of plant-based replacements for plant-based spreads **(A)** and plant-based milk **(B)** (indicated as servings per week). Bars and whiskers indicate mean and standard deviation, respectively, for the visits t0 (baseline), t6 (after 6 months) and t12 (after twelve months). Green and blue bars depict frequencies in the WFPB and the DGE group, respectively. Horizontal bars indicate results (*p* = *p*-values) of the ANCOVAs calculated for t6 and t12 values, respectively, with t0 values serving as covariates in both comparisons.

A relevant increase occurred in both group’s consumption of plant-based spreads at t6 (WFPB: M ± SD: 0.06 ± 0.22; *p* = 0.002; DGE: M ± SD: 0.04 ± 0.20; *p* = 0.024). By t12, a further increase only occurred in the WFPB group (M ± SD: 0.18 ± 0.48; *p* < 0.001). This difference in consumption changes at t12 (*p* = 0.001; WFPB: M ± SD: 1.54 ± 3.55; DGE: M ± SD: 0.63 ± 1,32, Figure 4A), indicate a more pronounced adoption of plant-based spreads among the WFPB group over the course of the study.

For plant-based milk, participants in both the WFPB and DGE group maintained consistent, low intake levels throughout the study period, with no relevant changes observed (Figure 4B).

### 3.6 Neuroprotective food items

There was a difference observed in green tea consumption between the WFPB group and the DGE group (Figure 3A). Initially, the WFPB group increased their intake during the first six months (M ± SD: 0.18 ± 0.83; *p* = 0,015), in contrast to the DGE group, which reduced their intake (M ± SD: −0.05 ± 0.36*p* = 0.136). This trend continued into the observation phase, further increasing the distinction in consumption habits between the two groups (*p* = 0.004; WFPB: M ± SD: 0.38 ± 1.39; DGE: M ± SD: 0.18 ± 0.63at t6; *p* = 0.005; WFPB: M ± SD: 0.51 ± 1.60; DGE: M ± SD: 0.17 ± 0.41at t12).

Intake of unsalted nuts increased in both the WFPB and DGE groups throughout the study, with no statistically significant differences observed at t12 (Figure 3B). Herb, walnut and seed consumption also increased in both groups at t6 and continued to increase slightly at t12, with no significant differences between the groups (Figures 3C–E).

Regarding berries, leafy vegetables, broccoli, and mushrooms, intake levels were consistent across both groups throughout the study, showing minor and non-significant fluctuations (Figures 3F–I, respectively).

### 3.7 Fast food, snacks, sweets, sweet beverages, and alcohol

**Figure 5:**
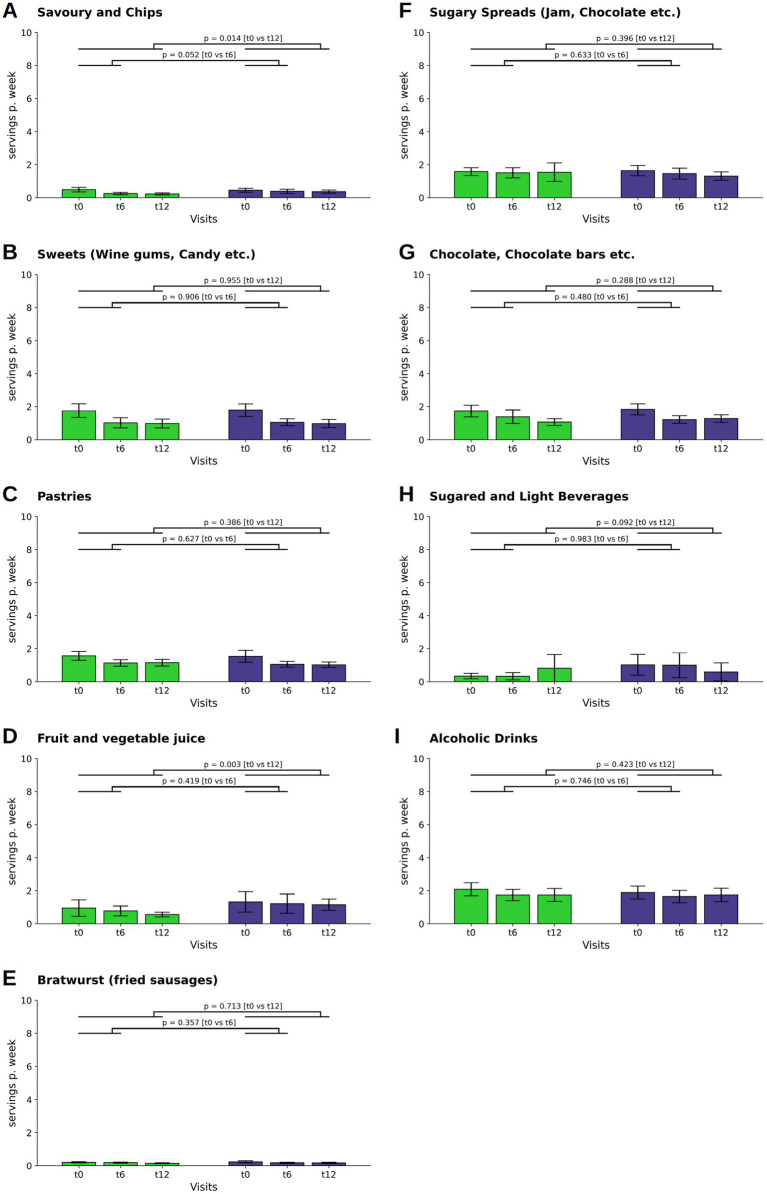
Changes in the consumption of savory snacks and chips **(A)**, sweets **(B)**, pastries **(C)**, fruit juice **(D)**, bratwurst **(E)**, sugary spreads **(F)**, chocolate and chocolate bars **(G)**, sugared and light beverages **(H)**, and alcoholic drinks **(I)** (depicted as servings per week). Bars and whiskers indicate mean and standard deviation, respectively, for the visits t0 (baseline), t6 (after 6 months) and t12 (after twelve months). Green and blue bars depict frequencies in the WFPB and the DGE group, respectively. Horizontal bars indicate results (*p* = *p*-values) of the ANCOVAs calculated for t6 and t12 values, respectively, with t0 values serving as covariates in both comparisons.

In the consumption of savory snacks and chips, the WFPB group experienced decline at both t6 and t12 (M ± SD: −0.03 ± 0.12; *p* = 0.002 at t6 and M ± SD: −0.04 ± 0.12; *p* = 0.001 at t12), and the groups demonstrated a difference at t12 (*p* = 0.014; WFPB: M ± SD: 0.03 ± 0.07; DGE: M ± SD: 0.05 ± 0.10, Figure 5A).

The category sweets as well as the category pastries saw reductions in consumption in both groups throughout the study period (sweets: WFPB: M ± SD: −0.11 ± 0.31; *p* < 0.001 at t6 and M ± SD: −0.11 ± 0.34; *p* < 0.001 at t12; DGE: M ± SD: −0.11 ± 0.30; *p* < 0.001 at t6 and M ± SD: −0.12 ± 0.37; *p* = 0.001 at t12. Pastries: WFPB: M ± SD: −0.06 ± 0.21; *p* = 0.001 at t6 and M ± SD: −0.06 ± 0.20; *p* = 0.001 at t12; DGE: M ± SD: −0.07 ± 0.31; *p* = 0.015 at t6 and M ± SD: −0.07 ± 0.34; *p* = 0.015 at t12), without statistically significant intergroup differences (Figures 5B,C).

For fruit and vegetable juice, the intergroup differences showed a reduction in the WFPB group (Figure 5D).

No significant intergroup changes were observed in the consumption of bratwurst, sugary spreads, chocolate and chocolate bars, sugared and light beverages, and alcoholic drinks across both groups throughout the study period (Figures 5E–I).

### 3.8 Frequency of cooking

At baseline 38.9% of the participants claimed to have not prepared a hot meal (lunch or dinner) from basic ingredients/fresh food themselves in the last 4 weeks. The frequency of cooking did not show any significant changes at both t6 and t12 across both WFPB and DGE groups.

### 3.9 Other parameters

The following additional food items and beverages were assessed during the study but are not presented in detail here, as they generally showed no statistically significant differences between the groups or exhibited minimal consumption levels: breakfast cereals; muesli; bread; pasta; cooked potatoes; fried potatoes; pizza; hamburger or doner kebab; cold cuts; fried animal foods; tofu; seitan; soy meat; margarine; olive oil; linola oil; dark chocolate; ice cream; fresh smoothies; water with calcium; black tea; coffee; sugar in coffee or tea; coffee or tea with milk, cream, or plant milk; red wine; use of supplements (for details see Supplementary Table 3).

## 4 Discussion

This study aimed to explore changes in dietary habits through online nutrition counselling among participants with MCI aged between 60 and 86 years. Participants initially did not meet recommended dietary guidelines, especially in their low intake of vegetables and fruits. While overall changes in dietary patterns were modest, there were several interesting developments. Both the WFPB and DGE groups demonstrated a slight increase in the consumption of whole grains and legumes. The WFPB group in particular showed a decrease in the intake of milk and dairy products. Additionally, the consumption of meat, fish, and eggs decreased slightly in both groups, with the WFPB group showing a slightly greater reduction. The intake of green tea and plant-based spreads showed a divergence, with the WFPB group substantially increasing their consumption. Conversely, the DGE group initially increased their intake of plant-based spreads from t0 to t6 but then reduced it from t6 to t12. Moreover, there was a mild increase in the consumption of berries and leafy greens and other neuroprotective foods like unsalted nuts and seeds, although these changes were not statistically significant.

Although the current study focuses on the impact of a purely plant-based whole-food diet on cognitive function, the observed reduction in egg consumption across both groups should not automatically be regarded as beneficial. In fact, several lines of evidence highlight potential cognitive advantages associated with moderate egg intake among older adults. For example, findings from the Rancho Bernardo Cohort indicated that egg consumption did not negatively impact various cognitive domains in this population. Specifically, men experienced improvements in verbal episodic memory (71), while women showed a modest enhancement in semantic memory (72). Furthermore, other research suggests that a regular inclusion of eggs in the diet may lower the risk of developing dementia (73). Recent studies have also pointed out that eggs, which are rich in nutrients such as choline, omega-3 fatty acids, and lutein, are associated with a reduced risk of Alzheimer’s dementia and related neuropathology (74). Complementary observational research supports the idea that an adequate intake of choline, particularly when derived from eggs and wheat germ, may positively affect cognitive function in the elderly (75).

Compared to data from the NVS II (German: Nationale Verzehrsstudie II, national nutrition study), collected between 2005 and 2007 from German citizens aged 14 to 80, our data set reveals the following differences. The proportion of individuals who do not consume animal products was lower at baseline at 0.8% compared to 2,5% in the NVSII but increased to 2,3% in the WFPB group at t12. Additionally in our data set 2.3% of the WFPB participants indicated never eating meat at t0 whereas the NVSII estimated 4.3% of the German population to be vegetarian (76). By t12, this percentage had slightly increased to 3.8% in the WFPB group.

A representative sample of 6,933 individuals aged 18 to 79 from the “Study on the Health of Adults in Germany” (DEGS1), conducted from 2008 to 2011, confirmed the results of the NVS II further indicating that 4.3% of the population (6.1% of women, 2.5% of men) adhere to a vegetarian diet (77).

In the present study the consumption of meat and meat products was already lower at baseline compared to NVS II and DEGS1, yet on average all surveys surpass the DGE-recommended intake (76, 78).

Several factors could account for this observation. For individuals born shortly after the war (1940–45), meat and animal products were less available, influencing lifelong dietary habits (79). Later in the 1960s the consumption of meat increased again with the economic upturn and the development of the meat industry, big slaughterhouses and meat becoming a coveted status symbol, which might have also had an impact on consumer beliefs. The corona pandemic (due to precarious financial situations) as well as the general vegetarian-health trend might have led to a lower consumption of animal products. As the influences and motivations may vary, this field remains to be further explored.

Furthermore, it is suggested that a phenomenon known as “social desirability” might have led to under-reporting of meat and animal product consumption. Also, for example the representation of whole grain consumption is surprisingly high, leading to the conclusion, the amount might be overreported or misunderstood (“darker brown bread” vs. real whole grain). Currently there are no comparable data on whole grain consumption in Germany. Additionally, the selection criteria might have inadvertently included a more health-conscious cohort, influencing the observed results.

All studies showed average consumption of vegetables and fruits below the recommendations set by the DGE. The DEGS1 revealed that a significant portion of the German population aged 18 to 79 (*n* = 7,116) did not meet the DGE recommendations for vegetables and fruit consumption but showed a slight increase in fruit consumption compared to the NVS II survey. In the DEGS1 on average, women and men consumed 3.1 and 2.4 portions of fruits and vegetables per day, respectively (80). Only 15% of women and 7% of men met the recommended intake of 5 portions per day. Fruit consumption tended to increase up to the age of 60 to 69 years. At least 3 portions per day were consumed by 39% of women and 25% of men, with the proportion increasing with higher social status (80).

It is important to note that demographic differences between the studies, particularly in terms of age, gender, educational level, occupation, and health status, can significantly influence dietary habits and, consequently, the outcomes of the studies. Therefore, these factors must be carefully considered when interpreting and comparing results. Notably, the percentage of participants with a higher level of education in the current study was higher than in the others. Additionally, it is important to note that the data from NVS II as well as DEGS1 are relatively dated, having been collected over a decade ago, which may affect their current relevance and applicability.

Recent data obtained from the forsa nutrition report provides an updated perspective on dietary patterns within the German population (81). This representative survey encompassed 1,001 citizens aged 14 and above, conducted in May 2023, and aimed to explore various aspects including preferences in eating habits, frequency of consumption of specific food items, criteria for selecting food products, dining outside the home and perceptions of the importance of various nutrition policy goals. In the forsa survey, 2% of respondents indicated following a vegan diet, abstaining from all animal products. A further 8% identified themselves as vegetarians, consuming animal-derived products like eggs, dairy, or honey, while 46% considered themselves flexitarian, generally limiting meat intake but not excluding it. Among participants aged 60 years and older, 1% were vegan, 5% vegetarian, and 49% flexitarian. Additionally, in this age group, 75% reported daily or multiple daily intakes of fruits and vegetables, 59% consumed dairy products such as yogurt or cheese one or more times per day, 19% ate meat or sausages once or multiple times daily, and 1% consumed fish and seafood daily (81).

The Planetary Health Diet (PHD) established by the EAT-Lancet Commission, as outlined in their 2019 report, identifies the greatest deficiencies in the consumption of nuts, seeds, legumes, and whole grain products. The EAT-Lancet Report offers a comprehensive framework designed to support a sustainable diet, aiming to minimize environmental damage while ensuring sufficient, safe, and nutritious food for the growing global population. The PHD recommends a reduction in the consumption of red meat and sugar by more than 50%, and double the intake of nuts, seeds, legumes, whole grains, vegetables, and fruits (18). This underlines the need for a strategic application of established dietary guidelines.

In our study nut and seed consumption as well as whole grain consumption saw an increase in intake in both groups after counselling throughout the intervention and the observational period without relevant intergroup differences. Legume intake had increased slightly by the 6-month mark in both the WFPB group and DGE group and stabilized at a similar level in both groups by the end of the 12-month study period. Although there was no significant increase, a positive trend could be observed. Therefore, nutrition education courses could be instrumental in addressing this gap by providing clear guidance to the public (28). In several cohort studies, an association has been found between lifestyle interventions through education, as well as country-level policy changes, and a lower incidence of dementia (82).

An interesting finding was the extent to which participants, mainly due to traditional gender roles, relied on others for their meal preparations. It was observed that many spouses accompanied participants during the course, indicating that they were primarily responsible for cooking and dietary choices. This raises concerns regarding the accuracy of the reported dietary habits. Discrepancies might have been introduced due to the reliance on others regarding food choices, suggesting that some participants may not have been closely involved in their dietary decisions. Additionally, nearly a third of the participants (29.6%) in our study lived alone, which could have influenced their ability to adopt dietary changes. Individuals who prepare meals independently may experience greater autonomy in modifying their diet, while those who rely on a partner, family member, or caregiver for meal preparation may face additional challenges in implementing dietary modifications. This distinction is particularly relevant in the context of MCI, where cognitive difficulties may further impact the ability to plan and prepare meals. Future research should explore the influence of living arrangements on dietary adherence, as social and environmental factors may play a crucial role in maintaining long-term dietary changes. Analyzing dietary behavior in individuals living alone versus those living with others could provide insights into tailored strategies that support sustainable dietary modifications in this population.

Sustaining dietary changes beyond the intervention period remains a challenge in nutritional counseling. Future interventions could incorporate structured follow-up sessions to provide continued guidance and address potential barriers to maintaining dietary habits. Digital tools, such as reminders, mobile applications, or online platforms, may support adherence by facilitating meal planning and progress tracking. Additionally, peer support networks and group discussions could contribute to long-term adherence by promoting social engagement and accountability. Personalized goal-setting and behavioral coaching may further aid in integrating dietary modifications into daily routines. Future research should examine the effectiveness of these approaches in maintaining dietary changes in individuals with MCI.

### 4.1 Strengths and limitations

The study has several strengths, including a well-maintained gender balance within the sample and a robust randomization process. The participants underwent thorough pre-screening to ensure their eligibility for inclusion in the MCI sample. The use of REDCap ensured that participants completed all questions. Additionally, we utilized validated measurement instruments, such as the MoCA. Conversely, the sample has certain limitations. Its composition is primarily determined by participants’ involvement in the RCT, suggesting that if individuals were enlisted solely for a survey, the sample might have varied characteristics. Consequently, this sample does not typify the broader population of individuals with MCI. The participants in this study possessed higher-than-average educational levels, potentially indicating an enhanced capacity for health-conscious living.

The online delivery format of the nutritional counseling sessions is another strength by enhancing accessibility and convenience, providing a benefit in reaching a broader demographic, including older adults and individuals with mobility disabilities. This digital approach facilitated the participation of individuals who might otherwise have found it challenging to engage in due to logistical constraints and showed the potential of remote health interventions. However, the findings are also constrained by the digital context, as we could only engage individuals that were able to undergo remote examinations. Participation in the study necessitated access to technical equipment like laptops or personal computers equipped with cameras and microphones, along with a stable internet connection for attending the twelve online nutritional counseling sessions. Additionally, the limited familiarity with digital media among the older participants, leading to potential errors in data input. Nonetheless, the remote nature of the study enabled us to recruit a total of 270 participants from various regions in Germany.

Another strength is the adaptation of the FFQ for a specific population with MCI during nutritional counseling representing a pioneering approach. This adjustment aims at examining dietary patterns relevant to cognitive health, thus contributing insights into an underexplored area of research. But there are also some limitations related to the use of the FFQ. The retrospective nature of the FFQ may introduce biases such as recall and confirmation bias. The aggregation of food groups could potentially lead to either underestimation or overestimation of certain components. The inclusion of the “never” category in the questionnaire may have caused ambiguity, as some participants might have interpreted it as consuming specific foods less than once a month due to the absence of intermediate options. While the original FFQ was modified to include additional items relevant to a WFPB diet, neuroprotective foods, and specific food variants like whole grains, there is a possibility for misinterpretation. Instead of utilizing visual aids for portion sizes, as done in the original FFQ, this study provided descriptions and examples, which, although detailed, could have introduced errors in portion size estimations. Also, the seasonality of certain foods and the various times of the nutrition education groups might have influenced the results. Additionally, diet was never examined in isolation; it was always coupled with computerized cognitive training programs, as highlighted in the study design. This integration may further limit the interpretability of the data regarding the direct impact of diet alone.

A potential limitation of this study is the differential emphasis on neuroprotective foods between the two dietary intervention groups. The WFPB group was encouraged to include plant-based foods with known neuroprotective properties, such as green leafy vegetables, nuts, berries, and legumes. While these foods were not actively discouraged in the DGE group, they were not explicitly emphasized to the same extent. This could introduce a potential bias in dietary outcomes, as participants in the WFPB group may have been more inclined to incorporate these foods. However, both dietary interventions followed structured guidelines based on existing dietary recommendations, and our analysis primarily focused on overall dietary changes rather than direct cognitive effects. Future studies could mitigate this potential bias by ensuring that both groups receive equivalent education on neuroprotective foods while maintaining distinct dietary frameworks.

Another potential limitation is that attendance at counseling sessions varied among participants. While all individuals received the same educational materials, lower attendance may have affected engagement and adherence to dietary recommendations. Future interventions could consider personalized follow-up support to enhance long-term adherence.

## 5 Conclusion

This analysis provides insights into the dietary patterns of patients with MCI. The findings reflect a mild shift toward more plant-based diets in the WFPB group compared to the more consistent dietary patterns observed in the DGE group. The persistence of dietary changes, particularly in the consumption of whole grains and a reduction in animal-source foods, indicates a potential shift toward healthier eating habits.

Overall, while some progress was made in adapting to healthier dietary choices, the substantial gap in achieving recommended dietary guidelines suggests a need for continued efforts and tailored strategies to enhance dietary adherence in this population. This study underscores the challenges in altering dietary habits in older adults but also highlights future areas of intervention, such as increasing the intake of neuroprotective foods.

Future research will be essential to further elucidate the mechanisms through which dietary patterns influence cognitive decline and to explore the long-term effects of these dietary changes. In future projects, the adherence rate among participants to the recommended guidelines should be systematically considered as a meaningful endpoint from the outset. This would enable a clearer assessment of the intervention’s effectiveness in terms of compliance and facilitate a more robust evaluation of its impact. It will also be critical to investigate the scalability of such interventions and their effectiveness across different populations with varying dietary backgrounds and health statuses. This could ultimately lead to more targeted dietary recommendations for individuals with MCI, contributing to the broader goal of enhancing cognitive health in aging populations.

## Data Availability

The raw data supporting the conclusions of this article will be made available by the authors, without undue reservation, upon justified request.
