# Supplementary material for: Effects of nutritional counseling on dietary patterns in patients with mild cognitive impairment: insights from the BrainFit-Nutrition study
Source: Front Nutr. 2025 Apr 28;12:1536939. doi: 10.3389/fnut.2025.1536939 (PMC12066778; doi:10.3389/fnut.2025.1536939)
Supplement: Supplementary file 1 [file Table_1.DOCX]

Supplementary Material

**Supplementary table 1: Food frequency questionnaire (FFQ) BrainFit-Nutrition (Based on the original DEGS-FFQ in German, modified for specific additional questions and translated into English for this manuscript submission)**

**Food frequency questionnaire (FFQ) BrainFit-Nutrition ^1^**

We are delighted that you are taking part in our study!

Eating and drinking have a lot to do with health. To better understand these connections, we would like to ask you to complete this questionnaire.

You will be asked how often and in what quantities you have eaten different foods in the last four weeks. Please also think about meals that you have eaten outside the home (e.g. in a restaurant or canteen). Please only think about your diet in the last four weeks!

Please answer each question. If you are not sure, please estimate. Please check only one answer for each question.

You may not eat or drink certain things. If so, please check "never" and move on to the next question.

The quantities given are average quantities.

Example: You eat 1 whole grain roll in the morning and 3 slices of whole grain bread in the evening. Please check "2 times a day" and "2 slices" (the average) as the quantity.

Compliance with data protection and confidentiality are important to us. Therefore, please do not write your name or telephone number on the questionnaire.

If you have any questions about completing the questionnaire, please contact our study team directly. The staff will be happy to help you.

Thank you very much!

Your BrainFit-Nutrition study team

^1^ Based on the DEGS-FFQ, modified for specific questions

| Food group | Analysis | No. | Question | Explanation | Answer | DEGS question,  DEGS modified (Mod) or  self-designed question |
| --- | --- | --- | --- | --- | --- | --- |
| Fruit | Food (LM) | 1 | How often have you eaten fresh fruit (e.g. apple, banana)? |  | Never Please continue with question    1 time a month 1 time a day  2-3 times a month 2 times a day  1-2 times a week 3 times a day  3-4 times a week 4-5 times a day  5-6 times a week More than 5 times a day | DEGS |
|  | LM | 1a | When you eat fresh fruit, how much of it do you usually eat? | 1 piece is e.g. 1 apple or 1 banana. Bowl means a small dessert bowl of 150 ml with e.g. strawberries or cherries. | ½ piece or ½ bowl (or less)  1 piece or 1 bowl  2 pieces or 2 bowls  3 pieces or 3 bowls  4 pieces or 4 bowls (or more) | DEGS |
|  | BrainFood (BF) | 2 | How often have you eaten berries? |  | Never Please continue with question    1 time a month 1 time a day  2-3 times a month 2 times a day  1-2 times a week 3 times a day  3-4 times a week 4-5 times a day  5-6 times a week More than 5 times a day | Self-designed |
|  |  | 2a | When you eat berries, how much do you usually eat? | 1 portion corresponds to a filled hand. | ¼ portion (or less)  ½ portion  1 portion  2 portions  3 portions (or more) | Self-designed |
| Vegetables | LM | 3 | How often have you eaten raw vegetables? | This refers to vegetables and lettuce that can be eaten raw, such as lettuce or raw carrots, peppers, etc. | Never Please continue with question    1 time a month 1 time a day  2-3 times a month 2 times a day  1-2 times a week 3 times a day  3-4 times a week 4-5 times a day  5-6 times a week More than 5 times a day | DEGS |
|  | LM | 3a | When you eat raw vegetables, how much do you usually eat? | Portion see picture | ¼ portion (or less)  ½ portion  1 portion  2 portions  3 portions (or more) | DEGS |
|  | LM | 4 | How often have you eaten cooked vegetables? |  | Never Please continue with question    1 time a month 1 time a day  2-3 times a month 2 times a day  1-2 times a week 3 times a day  3-4 times a week 4-5 times a day  5-6 times a week More than 5 times a day | DEGS |
|  | LM | 4a | If you eat cooked vegetables, how much of them do you usually eat? | Portion see picture. | ¼ portion (or less)  ½ portion  1 portion  2 portions  3 portions (or more) | DEGS |
|  | LM | 4b | If you eat cooked vegetables, this is normal: |  | Purchased fresh (raw)  Frozen vegetables  Canned vegetables  I don't know | DEGS |
|  | BF | 5 | How often have you eaten dark green leafy vegetables? | These include spinach, chard, kale, leaf lettuce and nettle. | Never Please continue with question    1 time a month 1 time a day  2-3 times a month 2 times a day  1-2 times a week 3 times a day  3-4 times a week 4-5 times a day  5-6 times a week More than 5 times a day | Self-designed |
|  | BF | 6 | How often have you eaten broccoli? |  | Never Please continue with question    1 time a month 1 time a day  2-3 times a month 2 times a day  1-2 times a week 3 times a day  3-4 times a week 4-5 times a day  5-6 times a week More than 5 times a day | Self-designed |
| Mushrooms | BF | 6 | How often have you eaten edible mushrooms? |  | Never Please continue with question    1 time a month 1 time a day  2-3 times a month 2 times a day  1-2 times a week 3 times a day  3-4 times a week 4-5 times a day  5-6 times a week More than 5 times a day | Self-designed |
|  | BF | 6a | If you have eaten edible mushrooms, which species were they? |  | (List all the types of mushrooms you have eaten in the last 4 weeks) | Self-designed |
| Herbs and spices | BF | 7 | How often have you consumed spices or fresh herbs (culinary herbs)? | This refers to dry spices such as pepper, turmeric, paprika, Provençal herbs or fresh herbs such as basil, rosemary, dill, etc. | Never Please continue with question    1 time a month 1 time a day  2-3 times a month 2 times a day  1-2 times a week 3 times a day  3-4 times a week 4-5 times a day  5-6 times a week More than 5 times a day | Self-designed |
|  | BF | 7a | If you use spices or fresh herbs, how much do you usually eat? |  | ½ tablespoon (or less)  1 tablespoon (spread)  2 tablespoons (spread)  3 tablespoons (spread)  4 tablespoons (or more) | Self-designed |
|  | BF | 7b | Which spices or fresh herbs do you mainly or regularly use? |  | - Free - | Self-designed |
| Grain/potato products | LM | 8 | How often have you eaten breakfast cereals? | These include cornflakes, chocolate pops, nougat bits, etc. | Never Please continue with question    1 time a month 1 time a day  2-3 times a month 2 times a day  1-2 times a week 3 times a day  3-4 times a week 4-5 times a day  5-6 times a week More than 5 times a day | Mod |
|  | LM | 8a | When you eat breakfast cereals, how much do you usually eat? | Please state quantities without milk. This refers to a dessert bowl of 150 ml. | ¼ bowl (or less)  ½ Bowl  1 bowl  2 bowls  3 bowls (or more) | Mod |
|  | LM | 9 | How often have you eaten muesli? | This refers to cereal flakes (pure) such as oat flakes or cereal flakes with dried fruit (Bircher muesli, muesli mixes). | Never Please continue with question    1 time a month 1 time a day  2-3 times a month 2 times a day  1-2 times a week 3 times a day  3-4 times a week 4-5 times a day  5-6 times a week More than 5 times a day | DEGS |
|  | LM | 9a | When you eat muesli, how much of it do you usually eat? | Please state quantities without milk. This refers to a dessert bowl of 150 ml. | ¼ bowl (or less)  ½ Bowl  1 bowl  2 bowls  3 bowls (or more) | DEGS |
|  | Whole grain (VK) | 9b | If you eat muesli, how often is it whole grain? |  | Rarely or never  About half  Predominantly  I don't know | Self-designed |
|  | LM | 10 | How often have you eaten bread or rolls? | This also refers to crispbread, toast, pretzels, etc. | Never Please continue with question    1 time a month 1 time a day  2-3 times a month 2 times a day  1-2 times a week 3 times a day  3-4 times a week 4-5 times a day  5-6 times a week More than 5 times a day | Self-designed |
|  | LM | 10a | When you eat bread or rolls, how much do you usually eat? |  | ½ slice or ½ roll (or less)  1 slice or 1 bread roll  2 slices or 2 rolls  3 slices or 3 rolls  4 slices (or more) | Self-designed |
|  | VK | 10b | When you eat bread or rolls, how often are they whole grain products? |  | Rarely or never  About half  Predominantly  I don't know | Self-designed |
|  | LM | 11 | How often have you eaten pasta (e.g. spaghetti, spaetzle, ravioli, lasagne)? |  | Never Please continue with question    1 time a month 1 time a day  2-3 times a month 2 times a day  1-2 times a week 3 times a day  3-4 times a week 4-5 times a day  5-6 times a week More than 5 times a day | DEGS |
|  | LM | 11a | When you eat pasta, how much of it do you usually eat? |  | ¼ plate (or less)  ½ Plate  1 plate  2 plates  3 plates (or more) | DEGS |
|  | VK | 11b | When you eat pasta, how often is it wholemeal pasta? |  | Rarely or never  About half  Predominantly  I don't know | Self-designed |
|  | BF | 11c | When you eat pasta, how often is it legume-based pasta? | This refers to pasta made from lentils, chickpeas or soy, for example. | Rarely or never  About half  Predominantly  I don't know | Self-designed |
|  | LM | 12 | How often have you eaten cooked grains? | These include rice, couscous, amaranth, millet, quinoa, bulgur, semolina, pearl barley, green spelt, barley, etc. | Never Please continue with question    1 time a month 1 time a day  2-3 times a month 2 times a day  1-2 times a week 3 times a day  3-4 times a week 4-5 times a day  5-6 times a week More than 5 times a day | Mod |
|  | LM | 12a | If you eat cooked grains, how much of them do you usually eat? | Portion see picture. | ¼ portion (or less)  ½ portion  1 portion  2 portions  3 portions (or more) | Mod |
|  | VK | 12b | If you eat cooked grains, how often are they whole grain products? | e.g. brown rice, wholegrain couscous | Rarely or never  About half  Predominantly  I don't know | Self-designed |
|  | LM | 13 | How often have you eaten boiled potatoes (e.g. boiled potatoes, jacket potatoes, potato dumplings)? |  | Never Please continue with question    1 time a month 1 time a day  2-3 times a month 2 times a day  1-2 times a week 3 times a day  3-4 times a week 4-5 times a day  5-6 times a week More than 5 times a day | DEGS |
|  | LM | 13a | When you eat boiled potatoes, how much do you usually eat? |  | ½ portion or 1 potato (or less)  1 portion or 2 potatoes  1 ½ portions or 3 potatoes  2 portions or 4 potatoes  2 ½ portions or 5 potatoes (or more) | DEGS |
|  | Junk Food (JF) | 14 | How often have you eaten potato products prepared in fat? | This refers to potato products such as croquettes, fried potatoes, pancakes or chips. | Never Please continue with question    1 time a month 1 time a day  2-3 times a month 2 times a day  1-2 times a week 3 times a day  3-4 times a week 4-5 times a day  5-6 times a week More than 5 times a day | Self-designed |
|  | JF | 14a | If you eat potato products prepared in fat, how much do you usually eat? |  | ¼ plate (or less)  ½ Plate  1 plate  2 plates  3 plates (or more) | Self-designed |
|  | JF | 15 | How often have you eaten pizza? | This also includes tarte flambée, pide etc. | Never Please continue with question    1 time a month 1 time a day  2-3 times a month 2 times a day  1-2 times a week 3 times a day  3-4 times a week 4-5 times a day  5-6 times a week More than 5 times a day | DEGS |
|  | JF | 15a | When you eat pizza, how much do you usually eat? | A portion is a frozen pizza of about 350 g. | ¼ portion (or less)  ½ portion  1 portion  2 portions  3 portions (or more) | DEGS |
| Dairy products | LM | 16 | How often have you drunk milk (including milk for coffee, cereal) in the last 4 weeks? | This refers to milk of animal origin, i.e. cow's milk, goat's milk, etc. | Never Please continue with question    1 time a month 1 time a day  2-3 times a month 2 times a day  1-2 times a week 3 times a day  3-4 times a week 4-5 times a day  5-6 times a week More than 5 times a day | Mod |
|  | LM | 16a | If you drink milk, how much do you usually drink? |  | 1/2 glass (or less)  1 glass (200ml)  2 glasses  3 glasses  4 glasses (or more) | Mod |
|  | LM | 16b | What kind of milk do you usually drink? |  | Whole milk (at least 3.5% fat))  Low-fat milk (1.5% fat)  Skimmed milk (max. 0.3% fat)  Soy ~~milk~~  Lactose-free milk  Other | DEGS |
|  | LM | 17 | How often have you eaten cream cheese? | This also includes cream cheese varieties such as herb cream cheese or cottage cheese.  This does NOT refer to vegan cream cheese alternatives. | Never Please continue with question    1 time a month 1 time a day  2-3 times a month 2 times a day  1-2 times a week 3 times a day  3-4 times a week 4-5 times a day  5-6 times a week More than 5 times a day | Mod |
|  | LM | 17a | When you eat cream cheese, how much of it do you usually eat? |  | ½ tablespoon (or less)  1 tablespoon (spread)  2 tablespoons (spread)  3 tablespoons (spread)  4 tablespoons (or more) | DEGS |
|  | LM | 18 | How often have you eaten cheese (soft, semi-hard or hard cheese)? | This does NOT refer to vegan cheese alternatives. Also think of sprinkled cheese for pizza etc. | Never Please continue with question    1 time a month 1 time a day  2-3 times a month 2 times a day  1-2 times a week 3 times a day  3-4 times a week 4-5 times a day  5-6 times a week More than 5 times a day | Mod |
|  | LM | 18a | When you eat cheese, how much do you usually eat? | See picture for slice or portion. | ½ slice or ½ portion (or less)  1 slice or 1 portion  2 slices or 2 portions  3 slices or 3 portions  4 slices or 4 portions (or more) | DEGS |
|  | LM | 19 | How often have you eaten quark, yogurt or soured milk? | This does NOT refer to vegan yogurt or quark alternatives | Never Please continue with question    1 time a month 1 time a day  2-3 times a month 2 times a day  1-2 times a week 3 times a day  3-4 times a week 4-5 times a day  5-6 times a week More than 5 times a day | Mod |
|  | LM | 19a | When you eat quark, yogurt or soured milk, how much of it do you usually eat? |  | ½ cup (or less)  1 cup (200 g)  2 cups  3 cups  4 cups | DEGS |
| Milk alternatives | Plantbased (pb) | 20 | How often did you drink plant-based drinks (including milk for coffee, cereal)? | This refers to all milk alternatives produced from plant-based sources such as soy milk, oat milk, nut milk, rice milk, etc. | Never Please continue with question    1 time a month 1 time a day  2-3 times a month 2 times a day  1-2 times a week 3 times a day  3-4 times a week 4-5 times a day  5-6 times a week More than 5 times a day | Self-designed |
|  | pb | 20a | If you drink plant-based drinks, how much do you usually drink? |  | 1/2 glass (or less)  1 glass (200ml)  2 glasses  3 glasses  4 glasses (or more) | Self-designed |
|  | pb | 20b | If you drink plant-based drinks, do they contain added vitamins or minerals or sugar? (multiple choice possible) |  | No additives  Vit B12  Vit D  Calcium  Sugar, agave syrup or similar. | Self-designed |
|  | JF | 21 | How often have you eaten plant-based (fresh) cheese alternatives (e.g. based on almond, cashew or coconut fat)? |  | Never Please continue with question    1 time a month 1 time a day  2-3 times a month 2 times a day  1-2 times a week 3 times a day  3-4 times a week 4-5 times a day  5-6 times a week More than 5 times a day | Self-designed |
|  | JF | 21a | When you eat (fresh) cheese, how much of it do you usually eat? | 1 portion of cream cheese is about 1 tablespoon | ½ slice or ½ portion (or less)  1 slice or 1 portion  2 slices or 2 portions  3 slices or 3 portions  4 slices or 4 portions (or more) | Self-designed |
| Meat, sausage, fish, egg | LM | 22 | How often have you eaten eggs? | This means, for example, boiled egg, scrambled egg, fried egg. | Never Please continue with question    1 time a month 1 time a day  2-3 times a month 2 times a day  1-2 times a week 3 times a day  3-4 times a week 4-5 times a day  5-6 times a week More than 5 times a day | DEGS |
|  | LM | 22a | When you eat eggs, how much do you usually eat? |  | ½ egg (or less)  1 egg  2 eggs  3 eggs  4 eggs (or more) | DEGS |
|  | LM | 23 | How often have you eaten poultry (e.g. chicken, chicken nuggets)? |  | Never Please continue with question    1 time a month 1 time a day  2-3 times a month 2 times a day  1-2 times a week 3 times a day  3-4 times a week 4-5 times a day  5-6 times a week More than 5 times a day | DEGS |
|  | LM | 23a | If you eat poultry, how much of it do you usually eat? |  | ¼ portion (or less)  ½ portion  1 portion  2 portions  3 portions (or more) | DEGS |
|  | JF | 24 | How often have you eaten hamburgers or doner kebabs? | This refers to animal-based hamburgers or doner kebabs. | Never Please continue with question    1 time a month 1 time a day  2-3 times a month 2 times a day  1-2 times a week 3 times a day  3-4 times a week 4-5 times a day  5-6 times a week More than 5 times a day | DEGS |
|  | JF | 24a | If you eat hamburgers or doner kebabs, how much  do you eat most of it? |  | ½ piece (or less)  1 piece  2 pieces  3 pieces  4 pieces (or more) | DEGS |
|  | JF | 25 | How often have you eaten bratwurst or currywurst? | This refers to bratwurst and currywurst of animal origin. | Never Please continue with question    1 time a month 1 time a day  2-3 times a month 2 times a day  1-2 times a week 3 times a day  3-4 times a week 4-5 times a day  5-6 times a week More than 5 times a day | DEGS |
|  | JF | 25a | When you eat bratwurst or currywurst, how much of it do you usually eat? |  | ½ piece (or less)  1 piece  2 pieces  3 pieces  4 pieces (or more) | DEGS |
|  | JF | 26 | How often have you eaten meat (e.g. pork, beef, game)? | This does not include sausage or poultry. | Never Please continue with question    1 time a month 1 time a day  2-3 times a month 2 times a day  1-2 times a week 3 times a day  3-4 times a week 4-5 times a day  5-6 times a week More than 5 times a day | DEGS |
|  | LM | 26a | When you eat meat, how much of it do you usually eat? | A portion is about 1 cutlet, 1 steak or 1 schnitzel; see picture. | ¼ portion (or less)  ½ portion  1 portion  2 portions  3 portions (or more) | DEGS |
|  | JF | 27 | How often have you eaten sausage (e.g. salami, liver sausage, ham)? |  | Never Please continue with question    1 time a month 1 time a day  2-3 times a month 2 times a day  1-2 times a week 3 times a day  3-4 times a week 4-5 times a day  5-6 times a week More than 5 times a day | Mod |
|  | JF | 27a | When you eat sausage, how much of it do you usually eat? |  | ½ disk  1 disk  2 slices  3 Slices  4 slices (or more) | DEGS |
|  | LM | 28 | How often have you eaten fish? | A portion means 1 fish fillet or 4 fish sticks. | Never Please continue with question    1 time a month 1 time a day  2-3 times a month 2 times a day  1-2 times a week 3 times a day  3-4 times a week 4-5 times a day  5-6 times a week More than 5 times a day | Mod |
|  | LM | 28a | When you eat fish, how much of it do you usually eat? |  | ¼ portion (or less)  ½ portion  1 portion  2 portions  3 portions (or more) | Mod |
|  | BF | 28b | If you eat fish, how often is it oily sea fish? | This includes herring, salmon, mackerel, eel and tuna. | (Almost) never  About ¼ of the consumption  About ½ of the consumption  About ¾ of the consumption  (Almost) always | Self-designed |
|  | JF | 29 | How often are the meat, poultry or fish products breaded or deep-fried? |  | (Almost) never  About ¼ of the consumption  About ½ of the consumption  About ¾ of the consumption  (Almost) always | DEGS |
| Protein-rich  foods | PB | 30 | How often have you eaten tofu or tempeh (in one piece, e.g. natural or smoked or with herbs)? | This does not include processed tofu products such as fried slices, tofu sausages or tofu schnitzel. | Never Please continue with question    1 time a month 1 time a day  2-3 times a month 2 times a day  1-2 times a week 3 times a day  3-4 times a week 4-5 times a day  5-6 times a week More than 5 times a day | Self-designed |
|  | PB | 30a | When you eat tofu or tempeh, how much of it do you usually eat? | A portion is about half of a standard 200g piece of tofu. | ¼ portion (or less)  ½ portion  1 portion  2 portions  3 portions (or more) | Self-designed |
|  | JF | 31 | How often have you eaten processed plant-based convenience products (meat, sausage or steak alternatives)? | This includes slices of meat, burger patties, sausages, schnitzel, steak made from soy, lupin or peas as well as plant-based sausage cold cuts or sausage spreads. | Never Please continue with question    1 time a month 1 time a day  2-3 times a month 2 times a day  1-2 times a week 3 times a day  3-4 times a week 4-5 times a day  5-6 times a week More than 5 times a day | Self-designed |
|  | JF | 31a | If you eat processed plant-based convenience foods, how much of them do you usually eat? | A portion means a sausage, a schnitzel, a slice of cold meat. | ¼ portion (or less)  ½ portion  1 portion  2 portions  3 portions (or more) | Self-designed |
|  | PB | 32 | How often have you eaten seitan products? | This refers to seitan products such as fried pieces (e.g. mock duck), seitan slices or seitan cold cuts. | Never Please continue with question    1 time a month 1 time a day  2-3 times a month 2 times a day  1-2 times a week 3 times a day  3-4 times a week 4-5 times a day  5-6 times a week More than 5 times a day | Self-designed |
|  | PB | 32a | If you eat seitan products, how much do you usually eat? | A portion means a seitan cutlet, a slice of cold meat, a piece of roast meat or similar. | ¼ portion (or less)  ½ portion  1 portion  2 portions  3 portions (or more) | Self-designed |
|  | PB | 33 | How often have you eaten texturized soy (soy granules, soy shreds)? | This does not include processed soy products such as chicken nuggets or burger patties. | Never Please continue with question    1 time a month 1 time a day  2-3 times a month 2 times a day  1-2 times a week 3 times a day  3-4 times a week 4-5 times a day  5-6 times a week More than 5 times a day | Self-designed |
|  | PB | 33a | If you eat texturized soy, how much of it do you usually eat? | A portion is about 1 handful of soy shreds or soy granules. | ¼ portion (or less)  ½ portion  1 portion  2 portions  3 portions (or more) | Self-designed |
|  | BF | 34 | How often have you eaten pulses (e.g. beans, peas, lentils)? | This does not refer to noodles, pastries or spreads based on pulses, but pulses as the main component, e.g. in lentil soup, as a side dish or as hummus. | Never Please continue with question    1 time a month 1 time a day  2-3 times a month 2 times a day  1-2 times a week 3 times a day  3-4 times a week 4-5 times a day  5-6 times a week More than 5 times a day | Mod |
|  | BF | 34a | If you eat pulses, how much of them do you usually eat? | Portion see picture. | ¼ portion (or less)  ½ portion  1 portion  2 portions  3 portions (or more) | DEGS |
|  |  | 35 | How often have you eaten plant-based spreads based on seeds, nuts or pulses? | This refers to vegetable spread creams such as tomato spread, lentil spread, etc. | Never Please continue with question    1 time a month 1 time a day  2-3 times a month 2 times a day  1-2 times a week 3 times a day  3-4 times a week 4-5 times a day  5-6 times a week More than 5 times a day | Self-designed |
|  |  | 35a | When you eat plant-based spreads, how much do you usually eat? |  | ½ tablespoon (or less)  1 tablespoon (spread)  2 tablespoons (spread)  3 tablespoons (spread)  4 tablespoons (or more) | Self-designed |
| animal and plant fats | LM | 36 | How often have you eaten butter (on bread etc.)? |  | Never Please continue with question    1 time a month 1 time a day  2-3 times a month 2 times a day  1-2 times a week 3 times a day  3-4 times a week 4-5 times a day  5-6 times a week More than 5 times a day | Mod |
|  | LM | 36a | When you eat butter, how much of it do you usually eat? |  | ½ teaspoon (or less)  1 teaspoon (spread)  2 teaspoons (spread)  3 teaspoons (spread)  4 teaspoons (or more) | Mod |
|  | LM | 37 | How often have you eaten margarine (on bread etc.)? |  | Never Please continue with question    1 time a month 1 time a day  2-3 times a month 2 times a day  1-2 times a week 3 times a day  3-4 times a week 4-5 times a day  5-6 times a week More than 5 times a day | Mod |
|  | LM | 37a | When you eat margarine, how much of it do you usually eat? |  | ½ teaspoon (or less)  1 teaspoon (spread)  2 teaspoons (spread)  3 teaspoons (spread)  4 teaspoons (or more) | Mod |
|  | BF | 38 | How often have you eaten unsalted nuts (e.g. peanuts, walnuts, hazelnuts)? | This also refers to unsweetened and unsalted nut butters, e.g. almond butter. | Never Please continue with question    1 time a month 1 time a day  2-3 times a month 2 times a day  1-2 times a week 3 times a day  3-4 times a week 4-5 times a day  5-6 times a week More than 5 times a day | DEGS |
|  | BF | 38a | If you eat unsalted nuts, how much do you usually eat? | Portion see picture. | ¼ portion (or less)  ½ portion  1 portion  2 portions  3 portions (or more) | DEGS |
|  |  | 38b | When you eat nuts, how often are they walnuts? |  | (Almost) never  About ¼ of the consumption  About ½ of the consumption  About ¾ of the consumption  (Almost) always | Self-designed |
|  | BF | 39 | How often have you eaten seeds and kernels (e.g. sunflower seeds, linseed, pumpkin seeds, sesame seeds)? | This also refers to seed mousses, e.g. tahini (made from sesame seeds). | Never Please continue with question    1 time a month 1 time a day  2-3 times a month 2 times a day  1-2 times a week 3 times a day  3-4 times a week 4-5 times a day  5-6 times a week More than 5 times a day | Self-designed |
|  | BF | 39a | If you eat seeds and kernels, how much of them do you usually eat? |  | ½ tablespoon (or less)  1 tablespoon (spread)  2 tablespoons (spread)  3 tablespoons (spread)  4 tablespoons (or more) | Self-designed |
|  | LM | 40 | How often have you used oils or fats? | This refers to olive oil, rapeseed oil, linseed oil or lard, butter, etc. | Never Please continue with question    1 time a month 1 time a day  2-3 times a month 2 times a day  1-2 times a week 3 times a day  3-4 times a week 4-5 times a day  5-6 times a week More than 5 times a day | Self-designed |
|  | LM | 40a | If you use oil or grease, how much do you use? |  | ½ tablespoon (or less)  1 tablespoon (spread)  2 tablespoons (spread)  3 tablespoons (spread)  4 tablespoons (or more) | Self-designed |
|  | LM | 40b | Which fat do you mainly use when preparing meat or fish or plant-based alternatives? |  | Butter  Margarine  Olive oil  Vegetable cooking fat (e.g. Biskin, Palmin)  Animal cooking fat (e.g. lard)  Sunflower oil, safflower oil, germ oil, rapeseed oil, etc.  I don't know  None | Mod |
|  | LM | 40c | Which fat do you mainly use when preparing  vegetables? |  | Butter  Margarine  Olive oil  Vegetable cooking fat (e.g. Biskin, Palmin)  Animal cooking fat (e.g. lard)  Sunflower oil, safflower oil, germ oil, rapeseed oil, etc.  I don't know  None | Mod |
|  |  | 40d | Which fat do you mainly use to prepare cold dishes? |  | Butter  Margarine  Olive oil  Vegetable cooking fat (e.g. Biskin, Palmin)  Animal cooking fat (e.g. lard)  Sunflower oil, safflower oil, germ oil, rapeseed oil, etc.  I don't know  None | Self-designed |
|  | BF | 41 | If you use oil, how often is this olive oil? |  | (Almost) never  About ¼ of the consumption  About ½ of the consumption  About ¾ of the consumption  (Almost) always | Self-designed |
|  | BF | 42 | If you use oil, how often is this linseed oil? |  | (Almost) never  About ¼ of the consumption  About ½ of the consumption  About ¾ of the consumption  (Almost) always | Self-designed |
| High-sugar LM, snacks | JF | 43 | How often have you eaten sweet spread? | This refers to jam, honey, nut nougat cream or syrup (e.g. maple syrup, agave syrup, sugar beet syrup), even if these are not eaten on bread. | Never Please continue with question    1 time a month 1 time a day  2-3 times a month 2 times a day  1-2 times a week 3 times a day  3-4 times a week 4-5 times a day  5-6 times a week More than 5 times a day | Mod. |
|  | JF | 44a | When you eat sweet spread, how much of it do you usually eat? |  | 1 teaspoon (or less)  2 teaspoons (heaped)  3 teaspoons (heaped)  4 teaspoons (heaped)  5 teaspoons (or more) | Mod |
|  | JF | 45 | How often have you eaten cakes, sweet pastries or tarts? |  | Never Please continue with question    1 time a month 1 time a day  2-3 times a month 2 times a day  1-2 times a week 3 times a day  3-4 times a week 4-5 times a day  5-6 times a week More than 5 times a day | DEGS |
|  | LM | 45a | When you eat cakes, sweet pastries or tarts, how much do you usually eat? | This also refers to cookies or waffles. | ½ piece (or less)  1 piece  2 pieces  3 pieces  4 pieces (or more) | DEGS |
|  | JF | 46 | How often have you eaten chocolate, chocolate bars (including chocolates)? | I'm not talking about dark chocolate. | Never Please continue with question    1 time a month 1 time a day  2-3 times a month 2 times a day  1-2 times a week 3 times a day  3-4 times a week 4-5 times a day  5-6 times a week More than 5 times a day | DEGS |
|  | JF | 46 | When you eat chocolate or chocolate bars, how much do you usually eat? |  | ½ small chocolate bar (or less)  ¼ bar or 1 small chocolate bar  ½ bar or 1 large chocolate bar  1 bar or 2 large chocolate bars  2 bars (or more) | Mod |
|  | BF | 47 | How often have you eaten dark chocolate |  | Never Please continue with question    1 time a month 1 time a day  2-3 times a month 2 times a day  1-2 times a week 3 times a day  3-4 times a week 4-5 times a day  5-6 times a week More than 5 times a day | Self-designed |
|  |  | 47a | When you eat dark chocolate, how much do you usually eat? |  | ¼ table (or less)  ½ blackboard  1 tablet  2 bars (or more) | Self-designed |
|  | JF | 48 | How often did you eat sweets? | This refers to sweets such as candies, gummy bears, licorice, etc. | Never Please continue with question    1 time a month 1 time a day  2-3 times a month 2 times a day  1-2 times a week 3 times a day  3-4 times a week 4-5 times a day  5-6 times a week More than 5 times a day | DEGS |
|  | JF | 48a | When you eat sweets, how much do you usually eat? |  | 1 piece  2-5 pieces  6-10 pieces  11-20 pieces  21 pieces (or more) | DEGS |
|  | JF | 49 | How often have you eaten ice cream? |  | Never Please continue with question    1 time a month 1 time a day  2-3 times a month 2 times a day  1-2 times a week 3 times a day  3-4 times a week 4-5 times a day  5-6 times a week More than 5 times a day | DEGS |
|  | JF | 49a | When you eat ice cream, how much do you usually eat? |  | ½ ball (or less)  1 ball  2 balls  3 balls  4 scoops (or more) | DEGS |
|  | JF | 49 | How often have you eaten salty snacks? | This refers to salty snacks such as potato potato chips, salted pretzels, crackers, salted nuts, savory snacks, etc. | Never Please continue with question    1 time a month 1 time a day  2-3 times a month 2 times a day  1-2 times a week 3 times a day  3-4 times a week 4-5 times a day  5-6 times a week More than 5 times a day | DEGS |
|  |  | 49a | When you eat salty snacks, how much do you usually eat? |  | ¼ bowl (or less)  ½ Bowl  1 bowl  2 bowls  3 bowls (or more) | DEGS |
| Drinks | JF | 50 | How often have you drunk sugary or low-calorie soft drinks (e.g. cola, Coke Zero lemonade, iced tea, malt beer, energy drinks)? |  | Never Please continue with question    1 time a month 1 time a day  2-3 times a month 2 times a day  1-2 times a week 3 times a day  3-4 times a week 4-5 times a day  5-6 times a week More than 5 times a day | DEGS |
|  | JF | 50a | If you drink sugary or low-calorie soft drinks, how much do you usually drink? |  | ½ glass (or less)  1 glass (200 ml)  2 glasses  3 glasses  4 glasses (or more) | DEGS |
|  | LM | 51 | How often did you drink fruit juices? |  | Never Please continue with question    1 time a month 1 time a day  2-3 times a month 2 times a day  1-2 times a week 3 times a day  3-4 times a week 4-5 times a day  5-6 times a week More than 5 times a day | Mod |
|  | LM | 51a | If you drink fruit juices, how much do you usually drink? |  | ½ glass (or less)  1 glass (200 ml)  2 glasses  3 glasses  4 glasses (or more) | DEGS |
|  | LM | 51b | If you drink fruit juices, how often are they homemade smoothies? | This refers to freshly prepared smoothies made from whole fruit and vegetables, e.g. spinach | (Almost) never  About ¼ of the consumption  About ½ of the consumption  About ¾ of the consumption  (Almost) always | Self-designed |
|  | LM | 52 | How often have you consumed calorie-free drinks? | Tap water, mineral water, flavored water (e.g. ginger water), fruit tea, herbal tea | Never Please continue with question    1 time a month 1 time a day  2-3 times a month 2 times a day  1-2 times a week 3 times a day  3-4 times a week 4-5 times a day  5-6 times a week More than 5 times a day | Self-designed |
|  | LM | 52a | If you drink calorie-free drinks, how much do you usually drink? |  | ½ glass (or less)  1 glass (200 ml)  2 glasses  3 glasses  4 glasses (or more) | Self-designed |
|  | PB | 52b | If you drink water, how often is it calcium water (>600 mg calcium per liter) |  | (Almost) never  About ¼ of the consumption  About ½ of the consumption  About ¾ of the consumption  (Almost) always | Self-designed |
|  | BF | 53 | How often did you drink black tea? |  | Never Please continue with question    1 time a month 1 time a day  2-3 times a month 2 times a day  1-2 times a week 3 times a day  3-4 times a week 4-5 times a day  5-6 times a week More than 5 times a day | Self-designed |
|  | BF | 53a | If you drink black tea, how much do you usually drink? |  | ½ cup (or less)  1 cup (150 ml)  2 cups  3 cups  4 cups (or more) | Self-designed |
|  | BF | 54 | How often did you drink green tea? |  | Never Please continue with question    1 time a month 1 time a day  2-3 times a month 2 times a day  1-2 times a week 3 times a day  3-4 times a week 4-5 times a day  5-6 times a week More than 5 times a day | Self-designed |
|  | BF | 54a | If you drink green tea, how much do you usually drink? |  | ½ cup (or less)  1 cup (150 ml)  2 cups  3 cups  4 cups (or more) | Self-designed |
|  | LM | 55 | How often did you drink coffee? |  | Never Please continue with question    1 time a month 1 time a day  2-3 times a month 2 times a day  1-2 times a week 3 times a day  3-4 times a week 4-5 times a day  5-6 times a week More than 5 times a day | DEGS |
|  | LM | 55a | When you drink coffee, how much do you usually drink? |  | ½ cup (or less)  1 cup  2 cups  3 cups  4 cups (or more) | DEGS |
|  | JF | 56 | Do you usually add sugar to your hot drinks? Sweeteners are not meant. | e.g. sugar or candy in tea, coffee | No  Yes, about 1 teaspoon per cup  Yes, 2 teaspoons per cup  Yes, 3 teaspoons (or more) per cup | DEGS |
|  |  | 57 | If you drink hot drinks, how often do you use milk, cream or plant-based drinks? | This refers to milk in coffee or black tea, for example. | (Almost) never  About ¼ of the consumption  About ½ of the consumption  About ¾ of the consumption  (Almost) always | Self-designed |
| Alcohol | JF | 58 | How often have you drunk alcoholic beverages? |  | Never Please continue with question    1 time a month 1 time a day  2-3 times a month 2 times a day  1-2 times a week 3 times a day  3-4 times a week 4-5 times a day  5-6 times a week More than 5 times a day | Mod |
|  |  | 58a | When you drink alcohol, how much do you usually drink? |  | ½ glass (or less)  1 glass (200 ml)  2 glasses  3 glasses  4 glasses (or more) | Mod |
|  |  | 59 | How often did you drink red wine? |  | Never Please continue with question    1 time a month 1 time a day  2-3 times a month 2 times a day  1-2 times a week 3 times a day  3-4 times a week 4-5 times a day  5-6 times a week More than 5 times a day | Self-designed |
|  |  | 59a | When you drink red wine, how much do you usually drink? |  | ½ glass (or less)  1 glass (200 ml)  2 glasses  3 glasses  4 glasses (or more) | Self-designed |
| Supplements |  | 60 | What supplements did you take? | This refers to all food supplements such as vitamins, minerals or herbal preparations that do not require a prescription.  Please state how often you take them and how they are dosed.  Please also consider DHA-enriched vegetable oils. | Free text | Self-designed |
|  |  | 61 | How often did you eat animal-based foods? |  | Never Please continue with question    1 time a month 1 time a day  2-3 times a month 2 times a day  1-2 times a week 3 times a day  3-4 times a week 4-5 times a day  5-6 times a week More than 5 times a day | Self-designed |
|  |  | 62 | How many times a week do you prepare a hot meal (lunch or dinner) yourself from basic ingredients/fresh food? |  | Daily  5-6 times per week  3-4 times a week  1-2 times per week  Never | DEGS |

*DEGS: German Health Interview and Examination Survey for Adults (Deutsche Gesundheitsstudie); Mod: Modified version of the original DEGS question; BF: BrainFit-specific question, self-designed for the study; LM: Food-related question from the DEGS or modified for the study; VK: Whole grain-related question (Vollkorn); PB: Plant-based food-related question; JF: Junk food-related question.*

**Supplementary table 2: Food items of the six food boxes**

| **Topic** | **WFPB** | **DGE** |
| --- | --- | --- |
| **Box 1: Basics 1** | Olive oil | Canola oil |
|  | Yeast flakes | Carrot pesto, dry mixture |
|  | Brown rise | Whole-grain pasta |
|  | Oat flakes | Muesli with oat flakes |
|  | Green tea | Herbal tea, mixed herbs |
|  | White beans, dried | Chickpea snack, roasted and salted |
| **Box 2: BrainFood / Healthy alternatives** | Walnut seeds | nut mixture |
|  | Blueberries (Vaccinium myrtillus), freeze-dried | Dried mango confection |
|  | Walnut oil | Linseed oil |
|  | Vegetable patties, dried mixture | Vegetable patties, dried mixture |
|  | Pumpkin seeds | Marinated almonds |
|  | Dark chocolate 70% | Chocolate sweetened with dates |
|  | Beluga lentils, dried | Brown basmati rice |
| **Box 3: Rich in protein and calcium** | Almond butter, unsweetened | Coconut blossom sugar |
|  | Smoked tofu | Vegetable stock, dried, salt-free |
|  | Whole-grain pasta | Ready-mix for whole-grain bread |
|  | Quinoa seeds | Couscous mixture with chickpeas and red lentils, dried |
|  | Tahini |  |
|  | Yellow lentils, dried | Bulgur |
|  | Linseed oil | Olive oil |
| **Box 4: Superfoods, herbs rich in phytochemicals** | Edible seed mixture | Sun flower seeds |
|  | Linseeds | Muesli rich in omega-3-fatty acids |
|  | Powdered curcuma | Herb mixture for sweet dishes |
|  | Mushrooms, dried | Sprouts mixture |
|  | Cacao powder, unsweetened | Linseed oil |
|  | Vegetable juice | Vegetable juice |
|  | Yellow millet | Pasta from spelt |
| **Box 5: Extras** | Red lentil pasta | Whole-grain pasta |
|  | Herbal tea, mixed herbs | Dried fruits confection with chocolate |
|  | Energy balls from dried fruits | Herbal tea, mixed herbs |
|  | Marinated tofu | Rice wafers with chocolate |
|  | Hemp oil | Oil mixture with herbs |
|  | Herb mixture for sweet dishes | Herbs mixture |
| **Box 6: Basics 2** | Olive oil | Linseed oil |
|  | Walnut seeds | Salted seeds |
|  | Brown rise with wild rise | Nut mixture with dried fruits |
|  | Whole-grain pasta with vegetable filling, dried | Ready- mix for spelt patties |
|  | Pumpkin pesto, dried mixture | Muesli with chocolate and nuts |
|  | Black beans, dried | Almond confection, sugarfree |

**Supplementary table 3: Dietary changes between the two groups (WFPB and DGE) over a 12-months period.**

|  |  | **t0** | | **t6** | | **ANCOVA at t6** | | | **t12** | | **ANCOVA at t12** | | |
| --- | --- | --- | --- | --- | --- | --- | --- | --- | --- | --- | --- | --- | --- |
| **Parameter** | **Measure / Frequency** | **WFPB Group** | **DGE Group** | **WFPB Group** | **DGE Group** | **F** | **p** | **eta2** | **WFPB Group** | **DGE Group** | **F** | **p** | **eta2** |
| Diet type | omnivore | 126 (95.5%) | 122 (94.6%) | 124 (93.9%) | 123 (95.3%) |  |  |  | 122 (92.4%) | 123 (95.3%) |  |  |  |
| Diet type | pescatarian | 2 (1.5%) | 5 (3.9%) | 2 (1.5%) | 4 (3.1%) |  |  |  | 2 (1.5%) | 4 (3.1%) |  |  |  |
| Diet type | ovolacto | 3 (2.3%) | 1 (0.8%) | 4 (3.0%) | 2 (1.6%) |  |  |  | 5 (3.8%) | 1 (0.8%) |  |  |  |
| Diet type | vegan | 1 (0.8%) | 1 (0.8%) | 2 (1.5%) | 0 (0.0%) |  |  |  | 3 (2.3%) | 1 (0.8%) |  |  |  |
| Fruits | portions p. day | 0.99±1.17 | 1.00±1.18 | 1.01±0.94 | 1.06±1.08 | 0.174 | 0.677 | 0.001 | 1.16±1.25 | 1.15±1.13 | 0.019 | 0.891 | <0.001 |
| Berries | portions p. day | 0.33±0.66 | 0.40±0.81 | 0.39±0.66 | 0.39±0.72 | 0.577 | 0.448 | 0.002 | 0.37±0.64 | 0.31±0.50 | 2.507 | 0.115 | 0.01 |
| Raw vegetables | portions p. day | 0.60±0.67 | 0.73±1.13 | 0.75±0.91 | 0.77±1.09 | 1.343 | 0.248 | 0.005 | 0.66±0.81 | 0.84±1.06 | 1.259 | 0.263 | 0.005 |
| Cooked vegetables | portions p. day | 0.71±0.67 | 0.69±0.58 | 0.79±0.70 | 0.77±0.76 | 0.001 | 0.971 | <0.001 | 0.83±0.62 | 0.77±0.72 | 0.581 | 0.446 | 0.002 |
| Kind of cooked vegetables | fresh foods | 117 (88.6%) | 98 (76.0%) | 90 (89.1%) | 82 (78.8%) |  |  |  | 75 (88.2%) | 80 (82.5%) |  |  |  |
| Kind of cooked vegetables | frozen foods | 11 (8.3%) | 19 (14.7%) | 9 (8.9%) | 16 (15.4%) |  |  |  | 7 (8.2%) | 14 (14.4%) |  |  |  |
| Kind of cooked vegetables | canned foods | 1 (0.8%) | 5 (3.9%) | 0 (0.0%) | 3 (2.9%) |  |  |  | 0 (0.0%) | 0 (0.0%) |  |  |  |
| Kind of cooked vegetables | I don't know | 2 (1.5%) | 4 (3.1%) | 1 (1.0%) | 1 (1.0%) |  |  |  | 3 (3.5%) | 3 (3.1%) |  |  |  |
| Leafy vegetables | times p. day | 0.26±0.27 | 0.26±0.28 | 0.32±0.35 | 0.30±0.33 | 0.488 | 0.486 | 0.002 | 0.30±0.25 | 0.31±0.31 | 0.052 | 0.82 | 0 |
| Broccoli | times p. day | 0.08±0.09 | 0.08±0.09 | 0.09±0.09 | 0.10±0.12 | 0.165 | 0.685 | 0.001 | 0.11±0.10 | 0.10±0.10 | 1.02 | 0.314 | 0.004 |
| Mushrooms | times p. day | 0.09±0.12 | 0.10±0.17 | 0.09±0.12 | 0.11±0.16 | 0.538 | 0.464 | 0.002 | 0.10±0.13 | 0.11±0.17 | 0.113 | 0.737 | <0.001 |
| Herbs | portions p. day | 0.37±0.41 | 0.35±0.41 | 0.47±0.54 | 0.46±0.57 | 0.107 | 0.744 | <0.001 | 0.49±0.56 | 0.53±0.76 | 0.442 | 0.507 | 0.002 |
| Breakfast cereals | portions p. day | 0.14±0.48 | 0.13±0.28 | 0.16±0.47 | 0.16±0.32 | 0.056 | 0.813 | <0.001 | 0.22±0.82 | 0.17±0.31 | 0.425 | 0.515 | 0.002 |
| Muesli | portions p. day | 0.33±0.40 | 0.30±0.47 | 0.37±0.42 | 0.39±0.49 | 1.712 | 0.192 | 0.007 | 0.48±0.85 | 0.51±1.12 | 0.398 | 0.528 | 0.002 |
| Whole-grain muesli | seldom | 5 (3.8%) | 8 (6.2%) | 5 (3.8%) | 3 (2.3%) |  |  |  | 3 (2.3%) | 2 (1.6%) |  |  |  |
| Whole-grain muesli | half/half | 16 (12.1%) | 23 (17.8%) | 14 (10.6%) | 25 (19.4%) |  |  |  | 18 (13.6%) | 28 (21.7%) |  |  |  |
| Whole-grain muesli | mostly | 70 (53.0%) | 53 (41.1%) | 82 (62.1%) | 75 (58.1%) |  |  |  | 86 (65.2%) | 78 (60.5%) |  |  |  |
| Whole-grain muesli | don't know | 8 (6.1%) | 2 (1.6%) | 5 (3.8%) | 1 (0.8%) |  |  |  | 3 (2.3%) | 1 (0.8%) |  |  |  |
| Whole-grain muesli | percentage | 52.61±37.40 | 45.00±37.65 | 59.24±35.64 | 59.57±33.94 | 2.931 | 0.088 | 0.011 | 62.88±33.36 | 62.60±31.72 | 1.228 | 0.269 | 0.005 |
| Bread | portions p. day | 1.29±1.49 | 1.40±1.37 | 1.11±1.24 | 1.03±0.98 | 2.18 | 0.141 | 0.008 | 0.95±1.13 | 1.12±1.19 | 0.923 | 0.338 | 0.004 |
| Whole-grain bread | seldom | 16 (12.1%) | 22 (17.1%) | 11 (8.3%) | 13 (10.1%) |  |  |  | 9 (6.8%) | 8 (6.2%) |  |  |  |
| Whole-grain bread | half/half | 50 (37.9%) | 48 (37.2%) | 43 (32.6%) | 39 (30.2%) |  |  |  | 42 (31.8%) | 39 (30.2%) |  |  |  |
| Whole-grain bread | mostly | 63 (47.7%) | 59 (45.7%) | 77 (58.3%) | 77 (59.7%) |  |  |  | 80 (60.6%) | 81 (62.8%) |  |  |  |
| Whole-grain bread | don't know | 0 (0.0%) | 0 (0.0%) | 0 (0.0%) | 0 (0.0%) |  |  |  | 0 (0.0%) | 1 (0.8%) |  |  |  |
| Whole-grain bread | Percentage | 61.33±25.82 | 60.04±25.95 | 67.12±23.38 | 67.36±23.61 | 0.274 | 0.601 | 0.001 | 68.45±22.55 | 69.53±21.83 | 0.657 | 0.418 | 0.003 |
| Pasta | portions p. day | 0.21±0.17 | 0.18±0.17 | 0.19±0.15 | 0.17±0.14 | 0.039 | 0.844 | <0.001 | 0.20±0.22 | 0.20±0.17 | 0.693 | 0.406 | 0.003 |
| Whole-grain pasta | seldom | 78 (59.1%) | 70 (54.3%) | 60 (45.5%) | 44 (34.1%) |  |  |  | 43 (32.6%) | 32 (24.8%) |  |  |  |
| Whole-grain pasta | half/half | 24 (18.2%) | 28 (21.7%) | 30 (22.7%) | 41 (31.8%) |  |  |  | 41 (31.1%) | 52 (40.3%) |  |  |  |
| Whole-grain pasta | mostly | 21 (15.9%) | 22 (17.1%) | 38 (28.8%) | 41 (31.8%) |  |  |  | 45 (34.1%) | 43 (33.3%) |  |  |  |
| Whole-grain pasta | don't know | 5 (3.8%) | 6 (4.7%) | 2 (1.5%) | 1 (0.8%) |  |  |  | 2 (1.5%) | 0 (0.0%) |  |  |  |
| Whole-grain pasta | percentage | 32.05±26.91 | 34.19±27.28 | 42.88±30.37 | 48.14±29.30 | 1.723 | 0.191 | 0.007 | 49.62±29.34 | 52.21±27.42 | 0.201 | 0.654 | 0.001 |
| Pasta from legumes | seldom | 113 (85.6%) | 106 (82.2%) | 104 (78.8%) | 102 (79.1%) |  |  |  | 102 (77.3%) | 103 (79.8%) |  |  |  |
| Pasta from legumes | half/half | 9 (6.8%) | 7 (5.4%) | 19 (14.4%) | 9 (7.0%) |  |  |  | 23 (17.4%) | 13 (10.1%) |  |  |  |
| Pasta from legumes | mostly | 3 (2.3%) | 3 (2.3%) | 3 (2.3%) | 7 (5.4%) |  |  |  | 2 (1.5%) | 5 (3.9%) |  |  |  |
| Pasta from legumes | don't know | 6 (4.5%) | 10 (7.8%) | 5 (3.8%) | 9 (7.0%) |  |  |  | 4 (3.0%) | 6 (4.7%) |  |  |  |
| Pasta from legumes | percentage | 18.86±13.53 | 18.18±13.30 | 21.52±15.77 | 21.01±17.92 | <0.001 | 0.994 | <0.001 | 22.05±15.54 | 21.01±16.82 | 0.13 | 0.719 | 0.001 |
| Grain or pseudograin | portions p. day | 0.10±0.15 | 0.12±0.18 | 0.13±0.19 | 0.12±0.18 | 1.322 | 0.251 | 0.005 | 0.16±0.38 | 0.16±0.22 | 0.398 | 0.528 | 0.002 |
| Whole-grain grain or pseudograin | seldom | 31 (23.5%) | 31 (24.0%) | 26 (19.7%) | 25 (19.4%) |  |  |  | 27 (20.5%) | 22 (17.1%) |  |  |  |
| Whole-grain grain or pseudograin | half/half | 33 (25.0%) | 31 (24.0%) | 30 (22.7%) | 33 (25.6%) |  |  |  | 29 (22.0%) | 34 (26.4%) |  |  |  |
| Whole-grain grain or pseudograin | mostly | 34 (25.8%) | 29 (22.5%) | 49 (37.1%) | 46 (35.7%) |  |  |  | 59 (44.7%) | 52 (40.3%) |  |  |  |
| Whole-grain grain or pseudograin | don't know | 6 (4.5%) | 10 (7.8%) | 6 (4.5%) | 6 (4.7%) |  |  |  | 5 (3.8%) | 9 (7.0%) |  |  |  |
| Whole-grain grain or pseudograin | percentage | 38.60±32.67 | 35.89±31.88 | 46.55±33.86 | 46.71±33.12 | 0.485 | 0.487 | 0.002 | 52.61±32.76 | 51.05±32.06 | <0.001 | 0.988 | <0.001 |
| Cooked potatoes | portions p. day | 0.32±0.27 | 0.34±0.29 | 0.26±0.21 | 0.28±0.27 | 0.579 | 0.447 | 0.002 | 0.28±0.25 | 0.30±0.27 | 0.005 | 0.941 | <0.001 |
| Fried potatoes | portions p. day | 0.07±0.09 | 0.08±0.10 | 0.06±0.08 | 0.06±0.09 | 0.34 | 0.561 | 0.001 | 0.06±0.08 | 0.06±0.08 | 0.024 | 0.876 | <0.001 |
| Pizza | portions p. day | 0.04±0.04 | 0.04±0.04 | 0.03±0.04 | 0.03±0.03 | 0.155 | 0.694 | 0.001 | 0.03±0.04 | 0.03±0.04 | 0.012 | 0.913 | <0.001 |
| Milk (of animal origin) | portions p. day | 0.41±0.72 | 0.43±0.62 | 0.26±0.42 | 0.34±0.40 | 2.665 | 0.104 | 0.01 | 0.23±0.35 | 0.36±0.41 | 9.282 | 0.003 | 0.035 |
| Kind of animal milk | whole milk | 49 (37.1%) | 55 (42.6%) | 26 (25.7%) | 34 (32.7%) |  |  |  | 23 (27.1%) | 31 (32.0%) |  |  |  |
| Kind of animal milk | skimmed milk (1,5% fat) | 51 (38.6%) | 32 (24.8%) | 33 (32.7%) | 39 (37.5%) |  |  |  | 29 (34.1%) | 33 (34.0%) |  |  |  |
| Kind of animal milk | other | 4 (3.0%) | 3 (2.3%) | 7 (6.9%) | 2 (1.9%) |  |  |  | 10 (11.8%) | 2 (2.1%) |  |  |  |
| Kind of animal milk | milk free from lactose | 2 (1.5%) | 5 (3.9%) | 1 (1.0%) | 2 (1.9%) |  |  |  | 1 (1.2%) | 4 (4.1%) |  |  |  |
| Kind of animal milk | skimmed milk (0,3% fat) | 1 (0.8%) | 1 (0.8%) | 2 (2.0%) | 1 (1.0%) |  |  |  | 2 (2.4%) | 1 (1.0%) |  |  |  |
| Cream cheese | times p. day | 0.29±0.51 | 0.30±0.36 | 0.27±0.48 | 0.28±0.34 | 0.061 | 0.805 | <0.001 | 0.21±0.31 | 0.32±0.38 | 7.564 | 0.006 | 0.028 |
| Cheese | portions p. day | 0.70±0.82 | 0.63±0.60 | 0.55±0.64 | 0.49±0.41 | 0.326 | 0.569 | 0.001 | 0.43±0.58 | 0.48±0.41 | 2.313 | 0.13 | 0.009 |
| Curd | portions p. day | 0.20±0.20 | 0.20±0.23 | 0.17±0.18 | 0.19±0.21 | 2.187 | 0.14 | 0.008 | 0.15±0.16 | 0.19±0.20 | 6.699 | 0.01 | 0.025 |
| Plant-based milk | portions p. day | 0.10±0.46 | 0.10±0.27 | 0.09±0.18 | 0.14±0.35 | 2.182 | 0.141 | 0.008 | 0.12±0.22 | 0.11±0.25 | 0.092 | 0.762 | <0.001 |
| Plant milk without supplements | occurrence | 30 (22.7%) | 37 (28.7%) | 41 (31.1%) | 44 (34.1%) |  |  |  | 52 (39.4%) | 43 (33.3%) |  |  |  |
| Plant milk with vitamin b12 | occurrence | 4 (3.0%) | 2 (1.6%) | 9 (6.8%) | 5 (3.9%) |  |  |  | 7 (5.3%) | 5 (3.9%) |  |  |  |
| Plant milk with vitamin d | occurrence | 4 (3.0%) | 3 (2.3%) | 7 (5.3%) | 5 (3.9%) |  |  |  | 7 (5.3%) | 4 (3.1%) |  |  |  |
| Plant milk with calcium | occurrence | 8 (6.1%) | 4 (3.1%) | 11 (8.3%) | 7 (5.4%) |  |  |  | 12 (9.1%) | 9 (7.0%) |  |  |  |
| Plant milk sugared | occurrence | 1 (0.8%) | 1 (0.8%) | 3 (2.3%) | 1 (0.8%) |  |  |  | 3 (2.3%) | 1 (0.8%) |  |  |  |
| Cheese from plant milk | portions p. day | 0.01±0.06 | 0.02±0.08 | 0.03±0.12 | 0.01±0.05 | 5.461 | 0.02 | 0.021 | 0.06±0.18 | 0.02±0.08 | 6.729 | 0.01 | 0.025 |
| Eggs | portions p. day | 0.19±0.23 | 0.14±0.14 | 0.16±0.22 | 0.14±0.15 | 5.103 | 0.025 | 0.019 | 0.13±0.15 | 0.16±0.16 | 14.352 | <0.001 | 0.053 |
| Poultry | portions p. day | 0.08±0.13 | 0.07±0.08 | 0.07±0.12 | 0.08±0.10 | 3.399 | 0.066 | 0.013 | 0.05±0.09 | 0.07±0.08 | 4.208 | 0.041 | 0.016 |
| Hamburger or doner kebab | times p. day | 0.01±0.03 | 0.02±0.03 | 0.01±0.03 | 0.01±0.02 | 1.203 | 0.274 | 0.005 | 0.01±0.02 | 0.01±0.03 | 4.073 | 0.045 | 0.016 |
| Bratwurst or currywurst (sausage) | portions p. day | 0.03±0.04 | 0.03±0.06 | 0.03±0.03 | 0.02±0.03 | 0.852 | 0.357 | 0.003 | 0.02±0.03 | 0.02±0.04 | 0.135 | 0.713 | 0.001 |
| Meat | times p. day | 0.21±0.18 | 0.21±0.20 | 0.15±0.14 | 0.19±0.19 | 7.317 | 0.007 | 0.028 | 0.16±0.29 | 0.17±0.17 | 0.191 | 0.662 | 0.001 |
| Cold cuts | times p. day | 0.42±0.44 | 0.35±0.39 | 0.31±0.41 | 0.29±0.37 | 1.733 | 0.189 | 0.007 | 0.28±0.39 | 0.26±0.30 | 1.217 | 0.271 | 0.005 |
| Fish | portions p. day | 0.15±0.13 | 0.15±0.14 | 0.14±0.13 | 0.16±0.16 | 3.798 | 0.052 | 0.015 | 0.14±0.14 | 0.18±0.17 | 5.078 | 0.025 | 0.019 |
| Kind of fish | seldom | 25 (18.9%) | 25 (19.4%) | 17 (12.9%) | 14 (10.9%) |  |  |  | 22 (16.7%) | 14 (10.9%) |  |  |  |
| Kind of fish | half/half | 51 (38.6%) | 49 (38.0%) | 50 (37.9%) | 58 (45.0%) |  |  |  | 46 (34.8%) | 57 (44.2%) |  |  |  |
| Kind of fish | mostly | 42 (31.8%) | 45 (34.9%) | 52 (39.4%) | 48 (37.2%) |  |  |  | 50 (37.9%) | 50 (38.8%) |  |  |  |
| Kind of fish | don't know | 2 (1.5%) | 4 (3.1%) | 3 (2.3%) | 4 (3.1%) |  |  |  | 5 (3.8%) | 4 (3.1%) |  |  |  |
| Kind of fish | percentage | 49.43±29.56 | 52.02±28.66 | 54.70±29.02 | 56.20±26.28 | 0.004 | 0.948 | <0.001 | 52.69±29.74 | 57.13±26.03 | 1.126 | 0.29 | 0.004 |
| Fried animal foods | seldom | 96 (72.7%) | 94 (72.9%) | 100 (75.8%) | 99 (76.7%) |  |  |  | 100 (75.8%) | 99 (76.7%) |  |  |  |
| Fried animal foods | half/half | 23 (17.4%) | 25 (19.4%) | 25 (18.9%) | 23 (17.8%) |  |  |  | 24 (18.2%) | 24 (18.6%) |  |  |  |
| Fried animal foods | mostly | 8 (6.1%) | 7 (5.4%) | 3 (2.3%) | 4 (3.1%) |  |  |  | 4 (3.0%) | 4 (3.1%) |  |  |  |
| Fried animal foods | don't know | 1 (0.8%) | 0 (0.0%) | 0 (0.0%) | 0 (0.0%) |  |  |  | 0 (0.0%) | 0 (0.0%) |  |  |  |
| Fried animal foods | percentage | 24.89±20.56 | 25.23±20.17 | 22.77±17.09 | 23.06±17.69 | 0.005 | 0.945 | <0.001 | 23.03±17.77 | 23.45±17.73 | 0.02 | 0.888 | <0.001 |
| Tofu | portions p. day | 0.01±0.03 | 0.02±0.09 | 0.02±0.05 | 0.02±0.08 | 0.298 | 0.586 | 0.001 | 0.04±0.07 | 0.02±0.09 | 6.658 | 0.01 | 0.025 |
| Meat alternatives | portions p. day | 0.05±0.26 | 0.04±0.20 | 0.06±0.24 | 0.03±0.11 | 3.27 | 0.072 | 0.013 | 0.05±0.16 | 0.03±0.11 | 2.438 | 0.12 | 0.009 |
| Seitan | portions p. day | 0.00±0.01 | 0.00±0.02 | 0.00±0.02 | 0.00±0.01 | 0.727 | 0.395 | 0.003 | 0.01±0.02 | 0.00±0.01 | 5.039 | 0.026 | 0.019 |
| Soy meat | portions p. day | 0.00±0.01 | 0.00±0.01 | 0.00±0.01 | 0.00±0.01 | 1.233 | 0.268 | 0.005 | 0.01±0.03 | 0.01±0.03 | 3.25 | 0.073 | 0.012 |
| Legumes | portions p. day | 0.16±0.32 | 0.10±0.12 | 0.22±0.32 | 0.16±0.23 | 1.136 | 0.288 | 0.004 | 0.21±0.24 | 0.18±0.26 | 0.043 | 0.836 | <0.001 |
| Plant-based spreads | portions p. week | 0.35±0.77 | 0.50±1.12 | 0.78±1.62 | 0.77±1,62 | 0.381 | 0.538 | 0.001 | 1.54±3.55 | 0.63±1,32 | 10.701 | 0.001 | 0.04 |
| Butter | portions p. day | 0.43±0.71 | 0.41±0.59 | 0.33±0.68 | 0.29±0.46 | 0.492 | 0.484 | 0.002 | 0.30±0.66 | 0.31±0.47 | 0.322 | 0.571 | 0.001 |
| Margarine | portions p. day | 0.15±0.49 | 0.09±0.25 | 0.16±0.43 | 0.08±0.23 | 1.304 | 0.254 | 0.005 | 0.10±0.21 | 0.08±0.24 | 0.106 | 0.745 | <0.001 |
| Nuts (unsalted) | portions p. day | 0.17±0.49 | 0.12±0.32 | 0.22±0.46 | 0.23±0.45 | 1.592 | 0.208 | 0.006 | 0.34±1.23 | 0.27±0.55 | 0.008 | 0.928 | <0.001 |
| Walnuts | seldom | 40 (30.3%) | 37 (28.7%) | 23 (17.4%) | 24 (18.6%) |  |  |  | 24 (18.2%) | 27 (20.9%) |  |  |  |
| Walnuts | half/half | 48 (36.4%) | 45 (34.9%) | 60 (45.5%) | 48 (37.2%) |  |  |  | 51 (38.6%) | 52 (40.3%) |  |  |  |
| Walnuts | mostly | 30 (22.7%) | 31 (24.0%) | 36 (27.3%) | 45 (34.9%) |  |  |  | 48 (36.4%) | 44 (34.1%) |  |  |  |
| Walnuts | don't know | 5 (3.8%) | 2 (1.6%) | 6 (4.5%) | 3 (2.3%) |  |  |  | 3 (2.3%) | 0 (0.0%) |  |  |  |
| Walnuts | percentage | 42.61±28.60 | 42.40±29.75 | 49.20±27.22 | 51.40±29.37 | 0.83 | 0.363 | 0.003 | 53.30±28.37 | 52.29±28.13 | 0.112 | 0.738 | <0.001 |
| Seeds | portions p. day | 0.23±0.46 | 0.19±0.31 | 0.33±0.53 | 0.23±0.34 | 1.998 | 0.159 | 0.008 | 0.33±0.52 | 0.28±0.61 | 0.077 | 0.782 | <0.001 |
| Animal and plant fats | times p. day | 0.89±0.58 | 0.78±0.47 | 0.91±0.64 | 0.87±0.53 | 0.443 | 0.506 | 0.002 | 0.98±0.72 | 0.97±0.65 | 0.498 | 0.481 | 0.002 |
| Kind of fats for meat | olive oil | 36 (27.3%) | 53 (41.1%) | 34 (33.7%) | 40 (38.5%) |  |  |  | 35 (41.2%) | 40 (41.2%) |  |  |  |
| Kind of fats for meat | plant oil not olive oil | 68 (51.5%) | 56 (43.4%) | 57 (56.4%) | 54 (51.9%) |  |  |  | 42 (49.4%) | 50 (51.5%) |  |  |  |
| Kind of fats for meat | butter | 11 (8.3%) | 3 (2.3%) | 5 (5.0%) | 1 (1.0%) |  |  |  | 2 (2.4%) | 1 (1.0%) |  |  |  |
| Kind of fats for meat | margarine | 4 (3.0%) | 9 (7.0%) | 0 (0.0%) | 4 (3.8%) |  |  |  | 2 (2.4%) | 3 (3.1%) |  |  |  |
| Kind of fats for meat | I don't know | 4 (3.0%) | 1 (0.8%) | 0 (0.0%) | 1 (1.0%) |  |  |  | 0 (0.0%) | 0 (0.0%) |  |  |  |
| Kind of fats for meat | none | 1 (0.8%) | 1 (0.8%) | 2 (2.0%) | 1 (1.0%) |  |  |  | 2 (2.4%) | 0 (0.0%) |  |  |  |
| Kind of fats for meat | fats for cooking of plant source | 7 (5.3%) | 5 (3.9%) | 2 (2.0%) | 3 (2.9%) |  |  |  | 2 (2.4%) | 2 (2.1%) |  |  |  |
| Kind of fats for meat | fats for cooking of animal source | 1 (0.8%) | 1 (0.8%) | 0 (0.0%) | 0 (0.0%) |  |  |  | 0 (0.0%) | 0 (0.0%) |  |  |  |
| Kind of fats for vegetables | olive oil | 47 (35.6%) | 62 (48.1%) | 36 (35.6%) | 48 (46.2%) |  |  |  | 37 (43.5%) | 46 (47.4%) |  |  |  |
| Kind of fats for vegetables | margarine | 6 (4.5%) | 7 (5.4%) | 1 (1.0%) | 5 (4.8%) |  |  |  | 2 (2.4%) | 3 (3.1%) |  |  |  |
| Kind of fats for vegetables | butter | 31 (23.5%) | 25 (19.4%) | 13 (12.9%) | 7 (6.7%) |  |  |  | 10 (11.8%) | 12 (12.4%) |  |  |  |
| Kind of fats for vegetables | plant oil not olive oil | 35 (26.5%) | 29 (22.5%) | 40 (39.6%) | 35 (33.7%) |  |  |  | 32 (37.6%) | 30 (30.9%) |  |  |  |
| Kind of fats for vegetables | I don't know | 4 (3.0%) | 3 (2.3%) | 1 (1.0%) | 2 (1.9%) |  |  |  | 1 (1.2%) | 1 (1.0%) |  |  |  |
| Kind of fats for vegetables | none | 7 (5.3%) | 1 (0.8%) | 7 (6.9%) | 5 (4.8%) |  |  |  | 3 (3.5%) | 1 (1.0%) |  |  |  |
| Kind of fats for vegetables | fats for cooking of animal source | 1 (0.8%) | 0 (0.0%) | 0 (0.0%) | 0 (0.0%) |  |  |  | 0 (0.0%) | 0 (0.0%) |  |  |  |
| Kind of fats for vegetables | fats for cooking of plant source | 1 (0.8%) | 2 (1.6%) | 1 (1.0%) | 2 (1.9%) |  |  |  | 0 (0.0%) | 2 (2.1%) |  |  |  |
| Kind of fats for cold dishes | olive oil | 74 (56.1%) | 77 (59.7%) | 67 (66.3%) | 74 (71.2%) |  |  |  | 62 (72.9%) | 72 (74.2%) |  |  |  |
| Kind of fats for cold dishes | plant oil not olive oil | 28 (21.2%) | 18 (14.0%) | 15 (14.9%) | 16 (15.4%) |  |  |  | 13 (15.3%) | 16 (16.5%) |  |  |  |
| Kind of fats for cold dishes | none | 3 (2.3%) | 8 (6.2%) | 4 (4.0%) | 4 (3.8%) |  |  |  | 1 (1.2%) | 2 (2.1%) |  |  |  |
| Kind of fats for cold dishes | I don't know | 3 (2.3%) | 3 (2.3%) | 2 (2.0%) | 1 (1.0%) |  |  |  | 2 (2.4%) | 1 (1.0%) |  |  |  |
| Kind of fats for cold dishes | butter | 19 (14.4%) | 20 (15.5%) | 8 (7.9%) | 7 (6.7%) |  |  |  | 4 (4.7%) | 1 (1.0%) |  |  |  |
| Kind of fats for cold dishes | margarine | 2 (1.5%) | 3 (2.3%) | 4 (4.0%) | 2 (1.9%) |  |  |  | 3 (3.5%) | 3 (3.1%) |  |  |  |
| Kind of fats for cold dishes | fats for cooking of plant source | 1 (0.8%) | 0 (0.0%) | 0 (0.0%) | 0 (0.0%) |  |  |  | 0 (0.0%) | 0 (0.0%) |  |  |  |
| Kind of fats for cold dishes | fats for cooking of animal source | 1 (0.8%) | 0 (0.0%) | 0 (0.0%) | 0 (0.0%) |  |  |  | 0 (0.0%) | 0 (0.0%) |  |  |  |
| Olive oil | seldom | 19 (14.4%) | 14 (10.9%) | 10 (7.6%) | 12 (9.3%) |  |  |  | 11 (8.3%) | 11 (8.5%) |  |  |  |
| Olive oil | half/half | 51 (38.6%) | 43 (33.3%) | 49 (37.1%) | 48 (37.2%) |  |  |  | 52 (39.4%) | 50 (38.8%) |  |  |  |
| Olive oil | mostly | 61 (46.2%) | 69 (53.5%) | 73 (55.3%) | 68 (52.7%) |  |  |  | 68 (51.5%) | 66 (51.2%) |  |  |  |
| Olive oil | don't know | 1 (0.8%) | 3 (2.3%) | 0 (0.0%) | 1 (0.8%) |  |  |  | 1 (0.8%) | 2 (1.6%) |  |  |  |
| Olive oil | percentage | 60.87±25.27 | 64.11±24.95 | 66.70±22.25 | 64.92±23.47 | 2.968 | 0.086 | 0.011 | 64.85±22.93 | 64.38±23.40 | 1.275 | 0.26 | 0.005 |
| Linola oil | seldom | 107 (81.1%) | 101 (78.3%) | 90 (68.2%) | 86 (66.7%) |  |  |  | 79 (59.8%) | 69 (53.5%) |  |  |  |
| Linola oil | half/half | 14 (10.6%) | 20 (15.5%) | 29 (22.0%) | 34 (26.4%) |  |  |  | 39 (29.5%) | 50 (38.8%) |  |  |  |
| Linola oil | mostly | 6 (4.5%) | 3 (2.3%) | 9 (6.8%) | 6 (4.7%) |  |  |  | 9 (6.8%) | 7 (5.4%) |  |  |  |
| Linola oil | don't know | 4 (3.0%) | 5 (3.9%) | 3 (2.3%) | 3 (2.3%) |  |  |  | 4 (3.0%) | 3 (2.3%) |  |  |  |
| Linola oil | percentage | 21.78±17.63 | 22.05±16.00 | 27.35±21.35 | 27.48±19.95 | 0.001 | 0.976 | <0.001 | 30.00±21.83 | 32.36±21.04 | 0.918 | 0.339 | 0.004 |
| Sugary spreads (jam, chocolate etc.) | portions p. day | 0.23±0.24 | 0.23±0.31 | 0.22±0.31 | 0.21±0.33 | 0.228 | 0.633 | 0.001 | 0.22±0.56 | 0.19±0.25 | 0.722 | 0.396 | 0.003 |
| Pastries | portions p. day | 0.22±0.27 | 0.22±0.36 | 0.16±0.20 | 0.15±0.18 | 0.237 | 0.627 | 0.001 | 0.16±0.20 | 0.15±0.17 | 0.755 | 0.386 | 0.003 |
| Chocolate, chocolate bars etc. | portions p. day | 0.25±0.36 | 0.26±0.33 | 0.20±0.41 | 0.17±0.23 | 0.501 | 0.48 | 0.002 | 0.15±0.21 | 0.18±0.23 | 1.135 | 0.288 | 0.004 |
| Dark chocolate | times p. day | 0.21±0.29 | 0.17±0.27 | 0.23±0.32 | 0.18±0.29 | 0.282 | 0.596 | 0.001 | 0.23±0.29 | 0.16±0.25 | 3.657 | 0.057 | 0.014 |
| Sweets (wine gums, candy etc.) | portions p. day | 0.25±0.42 | 0.26±0.38 | 0.15±0.31 | 0.15±0.21 | 0.014 | 0.906 | 0 | 0.14±0.27 | 0.14±0.25 | 0.003 | 0.955 | <0.001 |
| Ice cream | portions p. day | 0.15±0.23 | 0.13±0.19 | 0.11±0.15 | 0.11±0.14 | 0.132 | 0.717 | 0.001 | 0.08±0.14 | 0.08±0.14 | 0.158 | 0.691 | 0.001 |
| Savory and chips | portions p. day | 0.07±0.14 | 0.06±0.12 | 0.04±0.07 | 0.06±0.13 | 3.824 | 0.052 | 0.015 | 0.03±0.07 | 0.05±0.10 | 6.145 | 0.014 | 0.023 |
| Sugared and light beverages | portions p. day | 0.05±0.17 | 0.15±0.63 | 0.05±0.22 | 0.14±0.76 | <0.001 | 0.983 | 0 | 0.12±0.83 | 0.09±0.55 | 2.866 | 0.092 | 0.011 |
| Fruit juice | portions p. day | 0.14±0.50 | 0.19±0.62 | 0.11±0.30 | 0.17±0.59 | 0.654 | 0.419 | 0.003 | 0.08±0.14 | 0.16±0.34 | 8.849 | 0.003 | 0.033 |
| Fresh smoothies | seldom | 68 (51.5%) | 78 (60.5%) | 76 (57.6%) | 84 (65.1%) |  |  |  | 74 (56.1%) | 92 (71.3%) |  |  |  |
| Fresh smoothies | half/half | 7 (5.3%) | 5 (3.9%) | 7 (5.3%) | 7 (5.4%) |  |  |  | 12 (9.1%) | 8 (6.2%) |  |  |  |
| Fresh smoothies | mostly | 10 (7.6%) | 8 (6.2%) | 12 (9.1%) | 13 (10.1%) |  |  |  | 13 (9.8%) | 13 (10.1%) |  |  |  |
| Fresh smoothies | don't know | 0 (0.0%) | 0 (0.0%) | 0 (0.0%) | 0 (0.0%) |  |  |  | 0 (0.0%) | 1 (0.8%) |  |  |  |
| Fresh smoothies | percentage | 16.82±22.74 | 16.28±20.48 | 19.02±23.70 | 21.05±23.91 | 1.104 | 0.294 | 0.004 | 21.33±24.80 | 22.48±23.33 | 0.457 | 0.5 | 0.002 |
| Beverages without calories | portions p. day | 5.12±6.37 | 4.93±6.02 | 5.01±5.84 | 5.02±6.07 | 0.04 | 0.842 | <0.001 | 4.94±5.77 | 4.86±5.70 | <0.001 | 0.987 | <0.001 |
| Water with calcium | seldom | 71 (53.8%) | 67 (51.9%) | 65 (49.2%) | 53 (41.1%) |  |  |  | 66 (50.0%) | 60 (46.5%) |  |  |  |
| Water with calcium | half/half | 7 (5.3%) | 12 (9.3%) | 17 (12.9%) | 21 (16.3%) |  |  |  | 16 (12.1%) | 20 (15.5%) |  |  |  |
| Water with calcium | mostly | 13 (9.8%) | 12 (9.3%) | 18 (13.6%) | 13 (10.1%) |  |  |  | 16 (12.1%) | 14 (10.9%) |  |  |  |
| Water with calcium | don't know | 41 (31.1%) | 38 (29.5%) | 32 (24.2%) | 42 (32.6%) |  |  |  | 34 (25.8%) | 35 (27.1%) |  |  |  |
| Water with calcium | percentage | 23.75±21.78 | 24.77±21.86 | 29.05±25.16 | 27.75±23.12 | 0.849 | 0.358 | 0.003 | 27.73±24.18 | 28.02±23.59 | 0.031 | 0.86 | <0.001 |
| Black tea | portions p. day | 0.34±0.87 | 0.38±1.02 | 0.30±0.78 | 0.27±0.76 | 1.874 | 0.172 | 0.007 | 0.39±1.42 | 0.28±0.64 | 2.053 | 0.153 | 0.008 |
| Green tea | portions p. day | 0.20±0.71 | 0.23±0.80 | 0.38±1.39 | 0.18±0.63 | 8.66 | 0.004 | 0.032 | 0.51±1.60 | 0.17±0.41 | 8.001 | 0.005 | 0.03 |
| Coffee | portions p. day | 1.51±1.53 | 1.43±1.97 | 1.45±1.67 | 1.23±1.49 | 2.06 | 0.152 | 0.008 | 1.24±1.12 | 1.31±1.82 | 0.72 | 0.397 | 0.003 |
| Sugar in coffee or tea | no | 120 (90.9%) | 111 (86.0%) | 96 (95.0%) | 93 (89.4%) |  |  |  | 77 (90.6%) | 84 (86.6%) |  |  |  |
| Sugar in coffee or tea | 2 teaspoons | 2 (1.5%) | 1 (0.8%) | 0 (0.0%) | 0 (0.0%) |  |  |  | 0 (0.0%) | 0 (0.0%) |  |  |  |
| Sugar in coffee or tea | 1 teaspoon | 9 (6.8%) | 14 (10.9%) | 4 (4.0%) | 7 (6.7%) |  |  |  | 7 (8.2%) | 9 (9.3%) |  |  |  |
| Coffee or tea with milk, cream or plant milk | seldom | 68 (51.5%) | 51 (39.5%) | 73 (55.3%) | 61 (47.3%) |  |  |  | 80 (60.6%) | 65 (50.4%) |  |  |  |
| Coffee or tea with milk, cream or plant milk | half/half | 21 (15.9%) | 26 (20.2%) | 19 (14.4%) | 17 (13.2%) |  |  |  | 19 (14.4%) | 24 (18.6%) |  |  |  |
| Coffee or tea with milk, cream or plant milk | mostly | 40 (30.3%) | 46 (35.7%) | 37 (28.0%) | 45 (34.9%) |  |  |  | 30 (22.7%) | 36 (27.9%) |  |  |  |
| Coffee or tea with milk, cream or plant milk | don't know | 2 (1.5%) | 4 (3.1%) | 2 (1.5%) | 4 (3.1%) |  |  |  | 3 (2.3%) | 2 (1.6%) |  |  |  |
| Coffee or tea with milk, cream or plant milk | percentage | 41.67±31.27 | 46.78±31.55 | 39.55±30.92 | 43.80±32.45 | 0.061 | 0.806 | <0.001 | 35.95±29.29 | 40.81±30.66 | 0.342 | 0.559 | 0.001 |
| Alcoholic drinks | portions p. day | 0.30±0.40 | 0.27±0.39 | 0.25±0.34 | 0.23±0.38 | 0.105 | 0.746 | <0.001 | 0.25±0.39 | 0.25±0.41 | 0.643 | 0.423 | 0.002 |
| Red wine | portions p. day | 0.10±0.24 | 0.06±0.10 | 0.07±0.23 | 0.06±0.11 | 3.716 | 0.055 | 0.014 | 0.08±0.23 | 0.08±0.19 | 6.751 | 0.01 | 0.025 |
| Use of supplements | occurrence | 58 (43.9%) | 64 (49.6%) | 63 (47.7%) | 68 (52.7%) |  |  |  | 64 (48.5%) | 64 (49.6%) |  |  |  |
| Food from animal sources | times p. day | 1.08±0.88 | 1.07±0.96 | 0.82±0.79 | 0.88±0.81 | 0.515 | 0.474 | 0.002 | 0.78±0.90 | 0.86±0.73 | 0.875 | 0.35 | 0.003 |
| Frequency of cooking | times p. day | 0.08±0.14 | 0.09±0.13 | 0.08±0.14 | 0.07±0.11 | 2.674 | 0.103 | 0.01 | 0.07±0.13 | 0.07±0.13 | 0.156 | 0.693 | 0.001 |
| Whole grain use | seldom | 39 (29.5%) | 44 (34.1%) | 30 (22.7%) | 24 (18.6%) |  |  |  | 23 (17.4%) | 16 (12.4%) |  |  |  |
| Whole grain use | half/half | 57 (43.2%) | 52 (40.3%) | 40 (30.3%) | 42 (32.6%) |  |  |  | 37 (28.0%) | 49 (38.0%) |  |  |  |
| Whole grain use | mostly | 36 (27.3%) | 33 (25.6%) | 62 (47.0%) | 63 (48.8%) |  |  |  | 72 (54.5%) | 64 (49.6%) |  |  |  |
| Whole grain use | don't know | 0 (0.0%) | 0 (0.0%) | 0 (0.0%) | 0 (0.0%) |  |  |  | 0 (0.0%) | 0 (0.0%) |  |  |  |
| Whole grain use | percentage | 49.20±26.47 | 47.02±26.98 | 58.48±28.07 | 60.58±26.83 | 1.895 | 0.17 | 0.007 | 62.99±26.80 | 63.02±24.39 | 0.218 | 0.641 | 0.001 |
| Vegetables | portions p. day | 1.32±1.10 | 1.42±1.43 | 1.51±1.28 | 1.52±1.43 | 0.394 | 0.531 | 0.002 | 1.48±1.14 | 1.59±1.42 | 0.065 | 0.798 | <0.001 |
| Grains & potatoes | portions p. day | 2.47±1.67 | 2.56±1.62 | 2.26±1.46 | 2.21±1.26 | 0.865 | 0.353 | 0.003 | 2.35±2.08 | 2.50±1.71 | 0.262 | 0.609 | 0.001 |
| Milk & dairy products | portions p. day | 1.61±1.43 | 1.57±1.11 | 1.24±1.13 | 1.31±0.77 | 1.006 | 0.317 | 0.004 | 1.01±0.97 | 1.35±0.79 | 16.032 | <0.001 | 0.059 |
| Meat, fish & eggs | portions p. day | 1.09±0.67 | 0.96±0.61 | 0.85±0.65 | 0.89±0.61 | 10.403 | 0.001 | 0.039 | 0.79±0.69 | 0.87±0.51 | 9.131 | 0.003 | 0.034 |
| Plant milk & cheese | portions p. day | 0.12±0.47 | 0.12±0.29 | 0.13±0.23 | 0.15±0.37 | 0.51 | 0.476 | 0.002 | 0.18±0.31 | 0.13±0.27 | 1.982 | 0.16 | 0.008 |

*The table shows for each food category the descriptive statistics for the two diets ("WFPB" and "DGE") at the timepoints t0, t6 and t12 in terms of means and standard deviations (M ± SD) as well as the outcomes of ANCOVAs testing the difference between the diet groups at timepoints t6 and t12 with the t0 data as covariates (F-value, p-value and eta-squared as an estimate of the effect sizes).*

**Supplementary table 4: Within-group changes across visits.**

|  |  |  | **Differences between t0 and t6** | | | | **Differences between t0 and t12** | | | |
| --- | --- | --- | --- | --- | --- | --- | --- | --- | --- | --- |
| **Parameter** | **Measure** | **Group** | **M  ±  SD** | **T** | **p** | **d** | **M  ±  SD** | **T** | **p** | **d** |
| Fruits | servings p. day | WFPB | 0.02±0.86 | 0,32 | 0,752 | 0,02 | 0.17±1.30 | 1,51 | 0,133 | 0,14 |
|  |  | DGE | 0.06±0.96 | 0,69 | 0,49 | 0,05 | 0.15±1.07 | 1,57 | 0,119 | 0,13 |
| Berries | servings p. day | WFPB | 0.07±0.30 | 2,52 | 0,013 | 0,1 | 0.04±0.44 | 1,07 | 0,286 | 0,06 |
|  |  | DGE | -0.01±0.73 | -0,09 | 0,925 | 0,01 | -0.09±0.78 | -1,36 | 0,177 | 0,14 |
| Raw vegetables | servings p. day | WFPB | 0.14±0.66 | 2,49 | 0,014 | 0,18 | 0.05±0.67 | 0,94 | 0,351 | 0,07 |
|  |  | DGE | 0.04±0.58 | 0,72 | 0,47 | 0,03 | 0.11±0.61 | 2,05 | 0,042 | 0,1 |
| Cooked vegetables | servings p. day | WFPB | 0.08±0.50 | 1,79 | 0,076 | 0,11 | 0.12±0.50 | 2,73 | 0,007 | 0,19 |
|  |  | DGE | 0.08±0.71 | 1,31 | 0,194 | 0,12 | 0.08±0.65 | 1,34 | 0,182 | 0,12 |
| Leafy vegetables | times per day | WFPB | 0.06±0.25 | 2,77 | 0,006 | 0,19 | 0.04±0.25 | 2,01 | 0,046 | 0,17 |
|  |  | DGE | 0.04±0.22 | 2,04 | 0,044 | 0,13 | 0.05±0.23 | 2,48 | 0,014 | 0,17 |
| Broccoli | times per day | WFPB | 0.01±0.08 | 2 | 0,048 | 0,17 | 0.03±0.10 | 4,15 | <0.001 | 0,36 |
|  |  | DGE | 0.02±0.09 | 2,53 | 0,012 | 0,19 | 0.02±0.09 | 3,07 | 0,003 | 0,25 |
| Mushrooms | times per day | WFPB | 0.00±0.07 | 0,73 | 0,467 | 0,04 | 0.01±0.08 | 1,44 | 0,153 | 0,08 |
|  |  | DGE | 0.01±0.11 | 0,89 | 0,376 | 0,05 | 0.01±0.13 | 0,99 | 0,326 | 0,07 |
| Herbs | servings p. day | WFPB | 0.10±0.42 | 2,66 | 0,009 | 0,2 | 0.12±0.49 | 2,9 | 0,004 | 0,25 |
|  |  | DGE | 0.12±0.44 | 3,06 | 0,003 | 0,24 | 0.18±0.70 | 2,9 | 0,004 | 0,29 |
| Breakfast cereals | servings p. day | WFPB | 0.02±0.29 | 0,72 | 0,473 | 0,04 | 0.08±0.90 | 1,02 | 0,308 | 0,12 |
|  |  | DGE | 0.03±0.30 | 1,09 | 0,277 | 0,1 | 0.04±0.21 | 1,99 | 0,049 | 0,12 |
| Muesli | servings p. day | WFPB | 0.03±0.29 | 1,29 | 0,2 | 0,08 | 0.14±0.82 | 1,99 | 0,049 | 0,21 |
|  |  | DGE | 0.09±0.37 | 2,81 | 0,006 | 0,19 | 0.21±0.94 | 2,54 | 0,012 | 0,24 |
| Whole-grain muesli | percentage | WFPB | 6.63±23.46 | 3,25 | 0,001 | 0,18 | 10.27±28.89 | 4,08 | <0.001 | 0,29 |
|  |  | DGE | 14.57±32.03 | 5,17 | <0.001 | 0,41 | 17.60±34.85 | 5,74 | <0.001 | 0,51 |
| Bread | servings p. day | WFPB | -0.18±0.92 | -2,29 | 0,024 | 0,13 | -0.34±1.27 | -3,05 | 0,003 | 0,26 |
|  |  | DGE | -0.37±1.02 | -4,08 | <0.001 | 0,31 | -0.28±0.88 | -3,64 | <0.001 | 0,22 |
| Whole-grain bread | percentage | WFPB | 5.80±19.42 | 3,43 | 0,001 | 0,24 | 7.12±22.78 | 3,59 | <0.001 | 0,29 |
|  |  | DGE | 7.33±18.39 | 4,52 | <0.001 | 0,3 | 9.50±19.93 | 5,41 | <0.001 | 0,4 |
| Pasta | servings p. day | WFPB | -0.03±0.14 | -2,12 | 0,036 | 0,16 | -0.02±0.21 | -0,97 | 0,334 | 0,09 |
|  |  | DGE | -0.01±0.13 | -0,77 | 0,445 | 0,06 | 0.01±0.16 | 0,95 | 0,345 | 0,08 |
| Whole-grain pasta | percentage | WFPB | 10.83±27.06 | 4,6 | <0.001 | 0,38 | 17.58±28.45 | 7,1 | <0.001 | 0,62 |
|  |  | DGE | 13.95±21.77 | 7,28 | <0.001 | 0,49 | 18.02±22.06 | 9,28 | <0.001 | 0,66 |
| Pasta from legumes | percentage | WFPB | 2.65±13.41 | 2,27 | 0,025 | 0,18 | 3.18±16.44 | 2,22 | 0,028 | 0,22 |
|  |  | DGE | 2.83±14.29 | 2,25 | 0,026 | 0,18 | 2.83±13.60 | 2,36 | 0,02 | 0,19 |
| Grain or pseudograin | servings p. day | WFPB | 0.03±0.16 | 1,98 | 0,05 | 0,16 | 0.06±0.34 | 2,01 | 0,047 | 0,21 |
|  |  | DGE | 0.00±0.15 | 0,05 | 0,963 | 0 | 0.04±0.17 | 2,45 | 0,016 | 0,18 |
| Whole-grain grain or pseudograin | percentage | WFPB | 7.95±26.57 | 3,44 | 0,001 | 0,24 | 14.02±29.88 | 5,39 | <0.001 | 0,43 |
|  |  | DGE | 10.81±25.17 | 4,88 | <0.001 | 0,33 | 15.16±28.31 | 6,08 | <0.001 | 0,47 |
| Cooked potatoes | servings p. day | WFPB | -0.07±0.22 | -3,54 | 0,001 | 0,28 | -0.04±0.22 | -2,09 | 0,039 | 0,15 |
|  |  | DGE | -0.06±0.23 | -2,9 | 0,004 | 0,21 | -0.05±0.23 | -2,43 | 0,016 | 0,17 |
| Fried potatoes | servings p. day | WFPB | -0.01±0.05 | -2,15 | 0,034 | 0,12 | -0.01±0.07 | -1,69 | 0,094 | 0,13 |
|  |  | DGE | -0.02±0.08 | -2,44 | 0,016 | 0,19 | -0.02±0.07 | -2,61 | 0,01 | 0,19 |
| Pizza | servings p. day | WFPB | -0.01±0.03 | -2,86 | 0,005 | 0,19 | -0.01±0.03 | -3,2 | 0,002 | 0,23 |
|  |  | DGE | -0.01±0.04 | -2,41 | 0,017 | 0,2 | -0.01±0.04 | -2,64 | 0,009 | 0,21 |
| Milk (of animal origin) | servings p. day | WFPB | -0.14±0.61 | -2,68 | 0,008 | 0,24 | -0.18±0.64 | -3,13 | 0,002 | 0,31 |
|  |  | DGE | -0.09±0.52 | -1,88 | 0,063 | 0,16 | -0.07±0.50 | -1,53 | 0,129 | 0,13 |
| Cream cheese | times per day | WFPB | -0.02±0.22 | -1,17 | 0,244 | 0,05 | -0.08±0.48 | -1,93 | 0,056 | 0,19 |
|  |  | DGE | -0.02±0.31 | -0,63 | 0,53 | 0,05 | 0.02±0.34 | 0,6 | 0,55 | 0,05 |
| Cheese | servings p. day | WFPB | -0.15±0.70 | -2,42 | 0,017 | 0,2 | -0.27±0.68 | -4,61 | <0.001 | 0,38 |
|  |  | DGE | -0.14±0.49 | -3,27 | 0,001 | 0,27 | -0.15±0.50 | -3,49 | 0,001 | 0,3 |
| Curd | servings p. day | WFPB | -0.04±0.14 | -2,89 | 0,005 | 0,19 | -0.06±0.14 | -4,7 | <0.001 | 0,32 |
|  |  | DGE | -0.01±0.14 | -1,08 | 0,284 | 0,06 | -0.02±0.18 | -0,97 | 0,334 | 0,07 |
| Plant-based milk | servings p. day | WFPB | -0.01±0.42 | -0,25 | 0,806 | 0,03 | 0.02±0.44 | 0,57 | 0,569 | 0,06 |
|  |  | DGE | 0.04±0.24 | 1,77 | 0,079 | 0,12 | 0.02±0.18 | 1,02 | 0,31 | 0,06 |
| Cheese from plant milk | servings p. day | WFPB | 0.02±0.10 | 2,54 | 0,012 | 0,23 | 0.05±0.17 | 3,16 | 0,002 | 0,33 |
|  |  | DGE | -0.00±0.06 | -0,48 | 0,629 | 0,04 | 0.00±0.10 | 0,12 | 0,907 | 0,01 |
| Eggs | servings p. day | WFPB | -0.04±0.10 | -4,16 | <0.001 | 0,16 | -0.06±0.15 | -4,54 | <0.001 | 0,31 |
|  |  | DGE | -0.00±0.10 | -0,2 | 0,841 | 0,01 | 0.01±0.12 | 1,24 | 0,215 | 0,09 |
| Poultry | servings p. day | WFPB | -0.01±0.09 | -1,5 | 0,136 | 0,09 | -0.02±0.10 | -2,69 | 0,008 | 0,21 |
|  |  | DGE | 0.01±0.07 | 1,38 | 0,169 | 0,1 | -0.00±0.07 | -0,08 | 0,934 | 0,01 |
| Hamburger or doner kebab | times per day | WFPB | 0.00±0.02 | 0,11 | 0,915 | 0,01 | -0.00±0.02 | -2,82 | 0,006 | 0,22 |
|  |  | DGE | -0.00±0.02 | -2 | 0,047 | 0,14 | -0.00±0.03 | -0,61 | 0,544 | 0,05 |
| Bratwurst (fried sausages) | servings p. day | WFPB | -0.00±0.03 | -1,3 | 0,197 | 0,09 | -0.01±0.03 | -3,58 | <0.001 | 0,24 |
|  |  | DGE | -0.01±0.05 | -2,06 | 0,042 | 0,19 | -0.01±0.05 | -2,19 | 0,03 | 0,18 |
| Meat | times per day | WFPB | -0.06±0.13 | -5,31 | <0.001 | 0,36 | -0.05±0.29 | -1,8 | 0,073 | 0,19 |
|  |  | DGE | -0.02±0.16 | -1,3 | 0,197 | 0,09 | -0.03±0.17 | -2,27 | 0,025 | 0,18 |
| Cold cuts | times per day | WFPB | -0.11±0.26 | -4,99 | <0.001 | 0,27 | -0.15±0.29 | -5,79 | <0.001 | 0,35 |
|  |  | DGE | -0.06±0.22 | -3,08 | 0,003 | 0,16 | -0.09±0.27 | -3,59 | <0.001 | 0,25 |
| Fish | servings p. day | WFPB | -0.01±0.09 | -1,18 | 0,24 | 0,07 | -0.01±0.13 | -0,76 | 0,446 | 0,07 |
|  |  | DGE | 0.01±0.12 | 1,43 | 0,155 | 0,1 | 0.03±0.14 | 2,19 | 0,031 | 0,17 |
| Kind of fish | percentage | WFPB | 5.27±18.44 | 3,28 | 0,001 | 0,18 | 3.26±26.78 | 1,4 | 0,165 | 0,11 |
|  |  | DGE | 4.19±26.49 | 1,79 | 0,075 | 0,15 | 5.12±27.95 | 2,08 | 0,04 | 0,19 |
| Fried animal foods | percentage | WFPB | -2.12±15.45 | -1,58 | 0,117 | 0,11 | -1.86±17.47 | -1,22 | 0,224 | 0,1 |
|  |  | DGE | -2.17±18.43 | -1,34 | 0,183 | 0,11 | -1.78±20.91 | -0,97 | 0,335 | 0,09 |
| Tofu | servings p. day | WFPB | 0.01±0.04 | 3,26 | 0,001 | 0,29 | 0.03±0.06 | 4,94 | <0.001 | 0,52 |
|  |  | DGE | 0.00±0.08 | 0,45 | 0,652 | 0,04 | 0.00±0.08 | 0,13 | 0,896 | 0,01 |
| Meat alternatives | servings p. day | WFPB | 0.01±0.07 | 1,11 | 0,269 | 0,03 | -0.00±0.18 | -0,14 | 0,885 | 0,01 |
|  |  | DGE | -0.01±0.18 | -0,91 | 0,364 | 0,09 | -0.01±0.15 | -1,14 | 0,256 | 0,09 |
| Seitan | servings p. day | WFPB | 0.00±0.02 | 1,3 | 0,197 | 0,14 | 0.00±0.02 | 3,11 | 0,002 | 0,33 |
|  |  | DGE | -0.00±0.01 | -0,12 | 0,902 | 0,01 | -0.00±0.02 | -0,39 | 0,694 | 0,03 |
| Soy meat | servings p. day | WFPB | 0.00±0.01 | 1,63 | 0,107 | 0,18 | 0.01±0.03 | 2,79 | 0,006 | 0,34 |
|  |  | DGE | -0.00±0.01 | -0,18 | 0,854 | 0,01 | 0.00±0.02 | 2,06 | 0,041 | 0,16 |
| Legumes | servings p. day | WFPB | 0.06±0.35 | 1,99 | 0,049 | 0,19 | 0.05±0.33 | 1,76 | 0,081 | 0,17 |
|  |  | DGE | 0.06±0.21 | 3,1 | 0,002 | 0,31 | 0.08±0.24 | 3,78 | <0.001 | 0,4 |
| Plant-based spreads | servings p. day | WFPB | 0.06±0.22 | 3,23 | 0,002 | 0,35 | 0.18±0.48 | 4,17 | <0.001 | 0,48 |
|  |  | DGE | 0.04±0.20 | 2,28 | 0,024 | 0,2 | 0.03±0.16 | 1,93 | 0,056 | 0,15 |
| Butter | servings p. day | WFPB | -0.10±0.35 | -3,29 | 0,001 | 0,14 | -0.13±0.39 | -3,97 | <0.001 | 0,2 |
|  |  | DGE | -0.12±0.36 | -3,82 | <0.001 | 0,23 | -0.10±0.46 | -2,52 | 0,013 | 0,19 |
| Margarine | servings p. day | WFPB | 0.01±0.39 | 0,22 | 0,826 | 0,02 | -0.05±0.44 | -1,38 | 0,169 | 0,14 |
|  |  | DGE | -0.00±0.15 | -0,19 | 0,846 | 0,01 | -0.00±0.12 | -0,23 | 0,815 | 0,01 |
| Nuts (unsalted) | servings p. day | WFPB | 0.05±0.31 | 1,93 | 0,055 | 0,11 | 0.17±1.10 | 1,78 | 0,077 | 0,18 |
|  |  | DGE | 0.11±0.30 | 4,01 | <0.001 | 0,28 | 0.15±0.41 | 4,17 | <0.001 | 0,34 |
| Walnuts | percentage | WFPB | 6.59±19.95 | 3,8 | <0.001 | 0,24 | 10.68±23.48 | 5,23 | <0.001 | 0,37 |
|  |  | DGE | 8.99±25.45 | 4,01 | <0.001 | 0,3 | 9.88±23.19 | 4,84 | <0.001 | 0,34 |
| Seeds | servings p. day | WFPB | 0.09±0.43 | 2,47 | 0,015 | 0,19 | 0.10±0.48 | 2,31 | 0,023 | 0,2 |
|  |  | DGE | 0.05±0.30 | 1,72 | 0,088 | 0,14 | 0.10±0.59 | 1,87 | 0,064 | 0,2 |
| Animal and plant fats | times per day | WFPB | 0.03±0.50 | 0,59 | 0,558 | 0,04 | 0.10±0.62 | 1,77 | 0,079 | 0,15 |
|  |  | DGE | 0.09±0.41 | 2,49 | 0,014 | 0,18 | 0.19±0.64 | 3,4 | 0,001 | 0,34 |
| Olive oil | percentage | WFPB | 5.83±19.42 | 3,45 | 0,001 | 0,25 | 3.98±17.65 | 2,59 | 0,011 | 0,16 |
|  |  | DGE | 0.81±20.74 | 0,45 | 0,656 | 0,03 | 0.27±22.09 | 0,14 | 0,889 | 0,01 |
| Linola oil | percentage | WFPB | 5.57±18.26 | 3,5 | 0,001 | 0,28 | 8.22±18.28 | 5,17 | <0.001 | 0,41 |
|  |  | DGE | 5.43±16.63 | 3,71 | <0.001 | 0,3 | 10.31±20.24 | 5,79 | <0.001 | 0,55 |
| Sugary spreads (jam, chocolate etc.) | servings p. day | WFPB | -0.01±0.21 | -0,54 | 0,592 | 0,03 | -0.00±0.50 | -0,11 | 0,911 | 0,01 |
|  |  | DGE | -0.02±0.26 | -1,07 | 0,286 | 0,08 | -0.05±0.22 | -2,37 | 0,019 | 0,17 |
| Pastries | servings p. day | WFPB | -0.06±0.21 | -3,39 | 0,001 | 0,26 | -0.06±0.20 | -3,45 | 0,001 | 0,25 |
|  |  | DGE | -0.07±0.31 | -2,47 | 0,015 | 0,24 | -0.07±0.34 | -2,45 | 0,015 | 0,26 |
| Chocolate, chocolate bars etc. | servings p. day | WFPB | -0.05±0.46 | -1,23 | 0,222 | 0,13 | -0.09±0.30 | -3,67 | <0.001 | 0,33 |
|  |  | DGE | -0.09±0.35 | -2,88 | 0,005 | 0,31 | -0.08±0.32 | -2,83 | 0,005 | 0,28 |
| Dark chocolate | times per day | WFPB | 0.02±0.20 | 1,16 | 0,25 | 0,07 | 0.03±0.21 | 1,51 | 0,133 | 0,09 |
|  |  | DGE | 0.01±0.15 | 1,02 | 0,308 | 0,05 | -0.00±0.19 | -0,26 | 0,797 | 0,02 |
| Sweets (wine gums, candy etc.) | servings p. day | WFPB | -0.11±0.31 | -3,86 | <0.001 | 0,29 | -0.11±0.34 | -3,83 | <0.001 | 0,32 |
|  |  | DGE | -0.11±0.30 | -3,95 | <0.001 | 0,34 | -0.12±0.37 | -3,57 | 0,001 | 0,36 |
| Ice cream | servings p. day | WFPB | -0.04±0.18 | -2,56 | 0,012 | 0,21 | -0.07±0.23 | -3,45 | 0,001 | 0,36 |
|  |  | DGE | -0.03±0.14 | -2,13 | 0,035 | 0,16 | -0.05±0.16 | -3,69 | <0.001 | 0,31 |
| Savoury snacks and chips | servings p. day | WFPB | -0.03±0.12 | -3,11 | 0,002 | 0,3 | -0.04±0.12 | -3,46 | 0,001 | 0,34 |
|  |  | DGE | -0.01±0.11 | -1,01 | 0,316 | 0,08 | -0.01±0.09 | -1,62 | 0,107 | 0,12 |
| Sugared and light beverages | servings p. day | WFPB | -0.00±0.20 | -0,09 | 0,931 | 0,01 | 0.01±0.29 | 0,42 | 0,672 | 0,04 |
|  |  | DGE | -0.00±0.43 | -0,1 | 0,917 | 0,01 | -0.06±0.29 | -2,46 | 0,015 | 0,11 |
| Fruit and vegetable juice | servings p. day | WFPB | -0.02±0.54 | -0,5 | 0,619 | 0,06 | -0.06±0.43 | -1,48 | 0,141 | 0,15 |
|  |  | DGE | -0.02±0.51 | -0,34 | 0,734 | 0,03 | -0.02±0.39 | -0,72 | 0,474 | 0,05 |
| Fresh smoothies | percentage | WFPB | 2.20±19.17 | 1,32 | 0,19 | 0,09 | 4.51±18.91 | 2,74 | 0,007 | 0,19 |
|  |  | DGE | 4.77±20.05 | 2,7 | 0,008 | 0,21 | 6.20±19.85 | 3,55 | 0,001 | 0,28 |
| Beverages without calories | servings p. day | WFPB | -0.10±5.10 | -0,23 | 0,818 | 0,02 | -0.18±5.81 | -0,35 | 0,727 | 0,03 |
|  |  | DGE | 0.09±5.37 | 0,18 | 0,857 | 0,01 | -0.08±5.59 | -0,15 | 0,878 | 0,01 |
| Water with calcium | percentage | WFPB | 5.30±17.56 | 3,47 | 0,001 | 0,23 | 3.98±18.17 | 2,52 | 0,013 | 0,17 |
|  |  | DGE | 2.98±20.06 | 1,69 | 0,094 | 0,13 | 3.26±21.18 | 1,75 | 0,083 | 0,14 |
| bBlack tea | servings p. day | WFPB | -0.04±0.38 | -1,08 | 0,282 | 0,04 | 0.05±0.99 | 0,58 | 0,561 | 0,04 |
|  |  | DGE | -0.11±0.55 | -2,31 | 0,022 | 0,12 | -0.10±0.61 | -1,87 | 0,063 | 0,12 |
| Green tea | servings p. day | WFPB | 0.18±0.83 | 2,45 | 0,015 | 0,16 | 0.31±1.31 | 2,69 | 0,008 | 0,25 |
|  |  | DGE | -0.05±0.36 | -1,5 | 0,136 | 0,07 | -0.06±0.69 | -1,02 | 0,308 | 0,1 |
| Coffee | servings p. day | WFPB | -0.07±0.97 | -0,78 | 0,436 | 0,04 | -0.27±1.07 | -2,93 | 0,004 | 0,2 |
|  |  | DGE | -0.20±0.95 | -2,39 | 0,018 | 0,11 | -0.12±1.62 | -0,84 | 0,402 | 0,06 |
| Coffee or tea with milk, cream or plant milk | percentage | WFPB | -2.12±24.37 | -1 | 0,319 | 0,07 | -5.72±22.61 | -2,91 | 0,004 | 0,19 |
|  |  | DGE | -2.98±25.52 | -1,33 | 0,186 | 0,09 | -5.97±27.71 | -2,45 | 0,016 | 0,19 |
| Alcoholic drinks | servings p. day | WFPB | -0.05±0.23 | -2,45 | 0,015 | 0,13 | -0.05±0.27 | -2,09 | 0,038 | 0,12 |
|  |  | DGE | -0.04±0.19 | -2,09 | 0,039 | 0,09 | -0.02±0.21 | -1,12 | 0,263 | 0,05 |
| Red wine | servings p. day | WFPB | -0.02±0.08 | -3,03 | 0,003 | 0,09 | -0.02±0.09 | -2,29 | 0,023 | 0,07 |
|  |  | DGE | -0.00±0.05 | -0,54 | 0,593 | 0,02 | 0.02±0.15 | 1,79 | 0,076 | 0,15 |
| Food from animal sources | times per day | WFPB | -0.26±0.74 | -4,05 | <0.001 | 0,31 | -0.30±0.87 | -3,95 | <0.001 | 0,34 |
|  |  | DGE | -0.20±0.92 | -2,43 | 0,016 | 0,22 | -0.21±0.96 | -2,49 | 0,014 | 0,25 |
| Frequency of cooking | times per day | WFPB | -0.00±0.11 | -0,12 | 0,903 | 0,01 | -0.01±0.12 | -0,75 | 0,452 | 0,06 |
|  |  | DGE | -0.02±0.08 | -2,88 | 0,005 | 0,17 | -0.02±0.09 | -1,93 | 0,055 | 0,12 |
| Whole grain use | percentage | WFPB | 9.28±24.02 | 4,44 | <0.001 | 0,34 | 13.79±25.50 | 6,21 | <0.001 | 0,52 |
|  |  | DGE | 13.57±21.12 | 7,29 | <0.001 | 0,5 | 16.01±23.15 | 7,85 | <0.001 | 0,62 |
| Diet type | percentage | WFPB | 0.27±9.17 | 0,33 | 0,74 | 0,03 | 0.53±10.58 | 0,58 | 0,566 | 0,05 |
|  |  | DGE | -0.27±11.15 | -0,28 | 0,783 | 0,02 | -0.54±10.70 | -0,58 | 0,566 | 0,04 |
| Vegetables | servings p. day | WFPB | 0.20±0.93 | 2,44 | 0,016 | 0,16 | 0.17±0.95 | 2,04 | 0,044 | 0,15 |
|  |  | DGE | 0.10±0.98 | 1,19 | 0,238 | 0,07 | 0.17±1.02 | 1,86 | 0,066 | 0,12 |
| Grains & potatoes | servings p. day | WFPB | -0.21±1.01 | -2,39 | 0,018 | 0,13 | -0.12±2.15 | -0,65 | 0,516 | 0,06 |
|  |  | DGE | -0.35±1.21 | -3,25 | 0,001 | 0,24 | -0.05±1.36 | -0,43 | 0,665 | 0,03 |
| Milk & dairy products | servings p. day | WFPB | -0.37±1.06 | -3,99 | <0.001 | 0,29 | -0.60±1.20 | -5,73 | <0.001 | 0,49 |
|  |  | DGE | -0.26±0.89 | -3,29 | 0,001 | 0,27 | -0.22±0.87 | -2,85 | 0,005 | 0,23 |
| Meat, fish & eggs | servings p. day | WFPB | -0.24±0.40 | -6,86 | <0.001 | 0,36 | -0.30±0.54 | -6,38 | <0.001 | 0,44 |
|  |  | DGE | -0.07±0.35 | -2,27 | 0,025 | 0,12 | -0.09±0.41 | -2,58 | 0,011 | 0,17 |
| Plant milk & cheese | servings p. day | WFPB | 0.01±0.44 | 0,3 | 0,766 | 0,03 | 0.06±0.48 | 1,52 | 0,132 | 0,16 |
|  |  | DGE | 0.03±0.24 | 1,61 | 0,11 | 0,1 | 0.02±0.21 | 0,94 | 0,348 | 0,06 |

*The table shows for each food category and both diet groups ("WFPB" and "DGE") separately the immediate changes (differences between t0 and t6) and the long-term changes (between t0 and t12). For all changes, mean and standard deviation (M ± SD) as well as the outcomes of the paired t-test (T-*
*value, p-value and Cohen's d value) are presented.*
